# Developing a Digital Marketplace for Family Planning: Pilot Randomized Encouragement Trial

**DOI:** 10.2196/10756

**Published:** 2018-07-31

**Authors:** Eric P Green, Arun Augustine, Violet Naanyu, Anna-Karin Hess, Lulla Kiwinda

**Affiliations:** ^1^ Duke Global Health Institute Duke University Durham, NC United States; ^2^ Department of Behavioral Sciences School of Medicine, College of Health Sciences Moi University Eldoret Kenya

**Keywords:** family planning, unmet need, contraception, digital health, Kenya

## Abstract

**Background:**

Family planning is an effective tool for preventing death among women who do not want to become pregnant and has been shown to improve newborn health outcomes, advance women’s empowerment, and bring socioeconomic benefits through reductions in fertility and population growth. Yet among the populations that would benefit the most from family planning, uptake remains too low. The emergence of digital health tools has created new opportunities to strengthen health systems and promote behavior change. In this study, women with an unmet need for family planning in Western Kenya were randomized to receive an encouragement to try an automated investigational digital health intervention that promoted the uptake of family planning.

**Objective:**

The objectives of the pilot study were to explore the feasibility of a full-scale trial—in particular, the recruitment, encouragement, and follow-up data collection procedures—and to examine the preliminary effect of the intervention on contraception uptake.

**Methods:**

This pilot study tested the procedures for a randomized encouragement trial. We recruited 112 women with an unmet need for family planning from local markets in Western Kenya, conducted an eligibility screening, and randomized half of the women to receive an encouragement to try the investigational intervention. Four months after encouraging the treatment group, we conducted a follow-up survey with enrolled participants via short message service (SMS) text message.

**Results:**

The encouragement sent via SMS text messages to the treatment group led to differential rates of intervention uptake between the treatment and control groups; however, uptake by the treatment group was lower than anticipated (19/56, 33.9% vs 1/56, 1.8%, in the control group). Study attrition was also substantial. We obtained follow-up data from 44.6% (50/112) of enrolled participants. Among those in the treatment group who tried the intervention, the instrumental variables estimate of the local average treatment effect was an increase in the probability of contraceptive uptake of 41.0 percentage points (95% uncertainty interval −0.03 to 0.85).

**Conclusions:**

This randomized encouragement design and study protocol is feasible but requires modifications to the recruitment, encouragement, and follow-up data collection procedures.

**Trial Registration:**

ClinicalTrials.gov NCT03224390; https://clinicaltrials.gov/ct2/show/NCT03224390 (Archived by WebCite at http://www.webcitation.org/70yitdJu8)

## Introduction

Family planning is one of the most effective public health interventions, and more women than ever before are experiencing the benefits. Voluntary family planning has been shown to prevent maternal death among women who do not want to become pregnant [1], improve newborn health outcomes [2], and bring socioeconomic benefits through reductions in fertility and population growth [3]. Contraceptive use may also advance women’s empowerment, but the evidence is weak [4,5]. Globally, in 2017, an estimated 715 million married or in-union women of reproductive age were using a modern method of contraception (58%), an increase of 22% since 2000 [6].

Despite this positive trend, another 203 million women—primarily in Asia and Africa—want to prevent or delay pregnancy but are not using a modern method of contraception [6]. This situation is referred to as an *unmet need* for modern contraception, and it signals the presence of barriers to uptake that may include limited access to methods, concerns about side effects, and other issues such as cultural norms against use. The proportion of women with an unmet need for contraception is highest in Africa, where more than 46 million married and in-union women (22%) would like to prevent or delay childbirth but are not using a modern method of contraception.

In Kenya, for instance, 17% of currently married and in-union women of reproductive age [7] and 26% of sexually active unmarried women [8] have an unmet need for family planning. This translates into approximately 1.3 million women in the country who are not using contraception but say they would like to avoid pregnancy. Millions of others are either unaware of the potential benefits of contraception, misinformed about the full range of modern methods available, or unsatisfied with previous experiences using contraception [7].

In recognition of the needs of women and girls living in Kenya and beyond, a major international initiative called Family Planning 2020 launched at the London Summit on Family Planning with the goal of “expanding access to family planning information, services, and supplies to an additional 120 million women and girls in 69 of the world’s poorest countries by 2020.” This initiative has sparked important gains, but more work remains if this goal is to be realized. Since the launch of FP2020 in 2012, an additional 38.8 million women have begun using a modern method of contraception [9]. While this progress is above historic trends, it is substantially off the pace required to meet the goal of adding 120 million new users by 2020. This gap suggests the need for new approaches that can augment existing efforts to expand the coverage of family planning.

Traditionally, efforts to promote the uptake of family planning have focused on demand generation activities, supply-side activities, or a mixture of both. Demand generation interventions seek to change knowledge, attitudes, and practices regarding family planning. Common approaches include mass media advertising (also known as behavior change communication), one-on-one and small group discussions, and economic incentives such as conditional cash transfer programs. Supply-side interventions aim to increase access, improve quality, and lower costs for family planning services. A systematic review of 63 published evaluations of family planning interventions concluded that economic incentives and supply-side interventions had the most consistent effect on contraceptive use, but the overall quality of the evidence was low [10].

The emergence of digital health tools—such as short message service (SMS), interactive voice response, and mobile phone apps—have created new opportunities to strengthen health systems and promote behavior change [11,12], but the evidence base for digital health remains weak. As is the case for nondigital interventions [10], studies of digital health tools have found that it is easier to increase knowledge than to change behavior [13].

This pilot study represents another effort to promote behavior change through the use of an SMS text messaging intervention. Women with an unmet need for modern methods of contraception in Western Kenya were randomized to receive messages that encouraged them to try an investigational digital health intervention. The objectives of this pilot study were to explore the feasibility of a full-scale trial—in particular the recruitment, encouragement, and follow-up data collection procedures—and to examine the preliminary effect of the intervention on the uptake of contraception.

## Methods

### Recruitment

This was an external pilot study [14,15] conducted to inform the design and implementation of a separate full-scale trial. The study design was a randomized encouragement trial.

### Setting and Participants

The target population for this study was Kenyan women who had an unmet need for family planning, that is, women who were not using family planning but wished to delay or prevent pregnancy. The accessible population was limited to women with an unmet need living in Bungoma County, Kenya.

#### Recruitment and Eligibility Screening

Over a period of 4 weeks in 2017, from July 12 to August 6, we conducted recruitment exercises at 6 open-air markets throughout the county. We identified 21 market venues in Bungoma County and selected 5 large markets and 1 small market that maximized geographical coverage. We visited each market on its “market day,” the day of the week when foot traffic peaks. Market days for the selected markets were Sunday, Monday, Wednesday, Thursday, and Friday. Our team visited 2 markets on Fridays.

Our market stall advertised an opportunity to participate in the “Bungoma County Women’s Health Study.” A team of 4 female study team members, all Kenyan, staffed the study table and screened women for eligibility. To be eligible to enroll in the study, women had to (1) be between the ages of 18 and 35 years (inclusive), (2) have an unmet need for family planning, (3) live in Bungoma County, (4) demonstrate phone ownership, (5) opt-in to receiving calls and SMS text messages related to the study, (6) demonstrate basic ability to operate the study tablets, and (7) provide consent to participate in the study. Women who were pregnant or fewer than 4 months postpartum were excluded.

To begin the screening with an interested woman, a member of the study team asked the woman her age and county of residence. To demonstrate phone ownership and continue to the second stage of screening, the woman had to show the enumerator that she received a test SMS text message from the study shortcode. In the second phase of screening, the enumerator asked the woman if she was pregnant or currently using any method of family planning to prevent or delay pregnancy.

If the woman was eligible to move to the third stage of screening, the enumerator demonstrated how to use the tablet computer to complete the survey via audio computer assisted self-interview. The screening survey text and audio were available in English and Swahili. The woman had to demonstrate proficiency in an example exercise to continue to the full screening. Enumerators were on hand to assist participants who needed help using the tablet.

#### Unmet Need

In the third and final phase of screening, the woman completed the baseline survey to enable us to classify her unmet need status and to collect relevant background information. The baseline survey instrument included several modules from the 2014 Kenya Demographic and Health Survey (Phase 7, short form), including household characteristics, respondent’s background, reproduction, contraception, and marriage and sexual activity [7].

To define unmet need for this study, we followed guidelines published by the Demographic and Health Survey Program (DHS, revised 2012) [16] and other relevant scholarly reviews [17]. A woman was classified as having an unmet need if she reported no current use of contraception, was not identified by the survey as infecund, and said she did not want to be pregnant for at least 2 years. A woman could also be classified as having an unmet need if she was postpartum amenorrheic and reported that she did not want her last birth at all or wanted to become pregnant later than she did. We further classified women as having an unmet need for limiting (does not want to become pregnant at all) or spacing (wants to delay pregnancy for at least 2 years). We extended this classification of unmet need to women who were not married or in a union if they reported being sexually active in the past 6 months, thus, putting them at risk for pregnancy. See our Multimedia Appendices for survey questions (Appendix 1) and a detailed algorithm for determining unmet need (Appendix 2).

#### Enrollment

If a woman was eligible to participate in the study based on her responses to the screening, the tablet prompted the enumerator to review the informed consent form with her. If she consented to participate, the enumerator recorded her name and contact details in the study register. Every woman who completed the screening received an honorarium of KES 200 (approximately US $2) for her time and effort, regardless of whether she was eligible to participate in the study or consented to participate. Ineligible women were not informed about the specific reason that they were ineligible to prevent others from determining which answers would trigger eligibility. At the time of enrollment, we informed participants that we might invite them to learn more about family planning and women's health with one of our partners.

### Intervention

The investigational intervention was a digital health marketplace for family planning called Nivi [18]. At the time of the study, any woman (or man) in Bungoma County could send a toll-free SMS text message to the Nivi service to ask a question about reproductive health or trigger a free callback to complete an automated family planning counseling session via interactive voice response. This session resulted in a set of recommended methods that fit the client’s preferences and goals, along with referrals to local public and private providers offering one or more of these methods. After a period of time, clients were prompted to provide details about their experience with family planning providers and were eligible to receive a transportation voucher (approximately USD $2) as a nudge toward behavior change. The investigational intervention remained under active development during the pilot trial. Participants could text or call customer service representatives as needed.

### Experimental Design and Randomization

Since the service was available to anyone living in Bungoma County, it was not possible to restrict access and estimate the impact of the service through a randomized controlled trial. In situations like this, a randomized encouragement design can be very effective [19]. In a randomized encouragement design, participants are randomized to receive an invitation or special encouragement to receive an intervention. Not everyone who is encouraged will try the intervention (and some who are not invited will try it on their own); however, as long as those randomly assigned to receive the encouragement—“the treatment group”—try the intervention at a higher rate than those not encouraged—the “control group”—it is possible to estimate the impact of the intervention. This design has been used to study various interventions where two-sided noncompliance is possible [20-23].

In this pilot trial, we randomly allocated the sample of 112 enrolled women to the treatment or control arm (1:1). At the end of the recruitment period, the first author used the blockTools package [24] in R [25] to block randomize by age and baseline indicators of having attended postsecondary schooling, previous use and discontinuation of contraception, and being married or living in a union. One month after the end of the recruitment period, on October 2, 2017, women randomized to the encouragement arm received an invitation via SMS text message to try the service and complete a free family planning screening (plus bonus phone credit of approximately US $2, not conditional on the use of service). Women randomized to the control arm received a different set of messages thanking them for participating in the study; the control messages did not mention the investigational service.

### Outcome Data Collection

We conducted a follow-up survey between February 14 and March 13, 2018, approximately 4 months after we invited the treatment group to try the service. Participants could complete the survey for free via an SMS text message in their preferred language or choose to receive a free callback from a study enumerator to complete the survey over the phone. Any woman who attempted to send an SMS text message but experienced an error was flagged for enumerator follow-up. The study enumerator was blind to each participant’s assignment until the end of the survey. We sent up to 4 SMS text message reminders from our study shortcode (mask “DGHI”) to study participants who did not reply. Women who completed the survey received an honorarium of KES 200 (approximately US $2) to appreciate their time and effort.

The primary outcome under investigation was self-reported use of a modern method of contraception [26] since the baseline survey. This included women who adopted and subsequently discontinued a method during this period. The reference point for the start of the recall period was the national election conducted on August 8, 2017, several days after the end of the baseline survey. We obtained a binary indicator of attempted service use by querying the system logs for participant phone numbers. If a participant’s phone number was present in the system logs, we coded her as having tried the service.

### Statistical Analysis

Because encouragement designs lead to two-sided noncompliance, we planned to use instrumental variables regression to obtain an unbiased local average treatment effect (LATE) of the impact of service use on contraceptive uptake. We used the AER [27] package in R [25] to estimate LATE via two-stage least squares regression. In the first stage, we regressed the indicator of service use on the instrumental variable—a binary indicator of random assignment to the treatment group. In the second stage, we regressed the primary outcome of contraceptive uptake on the predicted values of service use from the first stage regression. Both regressions included baseline controls and the mode of follow-up survey. We used the ivpack [28] package to obtain corrected Huber-White SEs. The results of nonlinear specifications are presented in Multimedia Appendix 3.

For this approach to be valid, the instrumental variable (or instrument) must meet 3 assumptions: (1) The instrument is randomly assigned (independence assumption), (2) the instrument increases use of the investigational intervention, and (3) the instrument only affects the outcome through use of the intervention (exclusion restriction) [29]. We satisfy the independence assumption (1) through the randomized design, and we demonstrate assumption (2) to be true empirically. There is not a direct test of the exclusion restriction (3), but it seems reasonable to assume this is met because the encouragement to try the service did not itself encourage women to adopt contraception or otherwise counsel them on the importance of family planning.

One aim of the study was to test the recruitment procedures and examine the potential for attrition. We based the target sample size for the full trial on the assumption that a sample size of 50 would be needed in an individually randomized trial (25 per arm) to detect a difference in contraception uptake of 30 percentage points between the control group (10%) and the treatment group (40%), given an alpha of 5%, power of 80%, and a one-tailed test. We increased this sample size estimate by a factor of 2.8 to account for the fact that only a subset of the treatment group was expected to uptake the intervention (70%) and that there would be a differential rate of service uptake in the control group that was not encouraged (10%). The inflation factor was 1/(0.7 − 0.1)^2^, producing an adjusted target sample size of 139 [30].

### Ethical Review

Institutional Review Boards at Duke University and Moi University reviewed and approved this study protocol. This pilot study is registered with ClinicalTrials.gov (NCT03224390).

## Results

### Participant Characteristics

**Figure 1:**
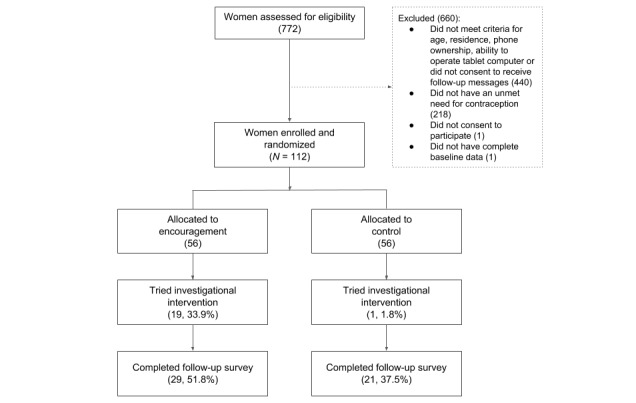
Participant flow diagram.

As shown in Figure 1, we assessed 772 women for eligibility and enrolled 112 women. A total of 660 women were excluded because they did not meet the inclusion criteria; 33.0% (218/660) of excluded women had a met need for contraception.

Table 1 summarizes the characteristics of the enrolled sample. The average age of participants was 24.7 (SD 4.8) years. The majority of women in the study were married or in a union, and two-thirds reported previous pregnancies. The average woman gave birth to 1.6 (SD 1.6) children and desired to have a total of 3.6 (SD 1.3) children. Most women reported an unmet need for spacing, rather than limiting. As is typical of women in Bungoma County, according to the most recent DHS, the women in this study were familiar with family planning methods. Most women indicated that they had recently been exposed to family planning messages in the media, and the average woman said she had heard of 9.6 (SD 2.2) out of 12 methods assessed.

### Intervention Uptake

The randomized encouragement design had only a modest effect on the probability of trying the intervention. Four months after the treatment group was encouraged via SMS text message to try the service, 33.9% (19/56) of women in the treatment group initiated a session, compared with only 1.8% (1/56) in the control group. The encouragement did produce a differential rate of uptake of 32.1 percentage points, but the difference was smaller than anticipated.

Table 2 shows the correlates of intervention use among the treatment group. Age was negatively associated with use, which was expected. No other baseline characteristics of participants were significantly associated with use.

**Table 1:** Participant characteristics.

Characteristic	Control (N=56)	Treatment (N=56)	Kenya Demographic and Health Survey Program 2014 Reference	
			Value	Reference Group
Age in years, mean (SD)	24.9 (4.6)	24.6 (5.0)	N/A^a^	N/A
Married or in union, %	55.4	60.7	59.7	All women, national, 20-24 years
Christian, %	96.4	94.6	91.4	All women, national, 15-49 years
Luhya tribe, %	75.0	78.6	15.0	All women, national, 15-49 years
Attended postsecondary schooling, %	19.6	17.9	7.2	All women, Bungoma, 15-49 years
No schooling, %	3.6	0.0	0.9	All women, Bungoma, 15-49 years
Nulligravida, %	30.4	33.9	35.3	All women, national, 20-24 years
Number of children born, mean (SD)	1.7 (1.6)	1.5 (1.6)	1.1^b^	All women, national, 20-24 years
Number of desired children, mean (SD)	3.7 (1.4)	3.4 (1.1)	3.6^b^	All women, national, 15-49 years
Unmet need for spacing, %	78.6	82.1	90.5	Currently married women^c^, national, 15-49 years
Past use of family planning, %	75.0	67.9	30.5	All women^d^, national, 15-49 years
Number methods known, mean (SD)^e^	9.7 (1.9)	9.4 (2.5)	8.7^b^	All women, national, 15-49 years
Not exposed to family planning messages, %^f^	21.4	17.9	18.9	All women, Western, 15-49 years

^a^N/A: not applicable.

^b^Standard deviation not reported.

^c^Currently married women with an unmet need for family planning.

^d^Women who started an episode of contraceptive use within the 5 years preceding the survey and discontinued within 12 months.

^e^Asked about knowledge of 12 different methods.

^f^Did not hear or see a family planning message on a radio or television or read in a newspaper or magazine in the past few months.

**Table 2:** Correlates of intervention uptake.

Characteristic^a^	Dependent variable (tried intervention)	*P* value
Age, beta (SE)	−.04 (.02)	.06
Married or in a union, beta (SE)	−.16 (.20)	.44
Identifies as Christian, beta (SE)	.44 (.33)	.20
Identifies as a member of Luhya tribe, beta (SE)	−.01 (.18)	.97
Attended postsecondary schooling, beta (SE)	.20 (.17)	.25
Nulligravida, beta (SE)	.09 (.24)	.71
Number of children born, beta (SE)	.04 (.10)	.65
Desired number of children, beta (SE)	.06 (.09)	.47
Has unmet need for spacing, beta (SE)	−.03 (.24)	.91
Past use of family planning, beta (SE)	−.26 (.17)	.14
Number of methods known, beta (SE)	.01 (.03)	.75
Not exposed to family planning messages, beta (SE)	−.19 (.20)	.35
Constant, beta (SE)	.71 (.58)	.23
Mean of dependent variable	.34	N/A^c^
Observations	56	N/A
*R* ^2^	.28	N/A
Adjusted *R*^2^	.08	N/A
Residual SE	.46_43_	N/A
*F* statistic	1.40_12,__43_	N/A

^a^Sample limited to women randomly assigned to the treatment group.

^b^Coefficients estimated through linear probability model regression.

^c^N/A: not applicable.

### Study Attrition

As shown in Figure 1, there was a substantial amount of attrition. We obtained follow-up data from 44.6% (50/112) of enrolled participants. Slightly more than half (56.0%, 28/50) of participants who completed the follow-up survey did so via an SMS text message (vs via a phone call with a study enumerator). Table 3 shows that attrition was higher among the control group, but this difference was not statistically significant. Attrition was significantly associated with a few baseline characteristics, including postsecondary education, nulligravida, and the mean number of children born; participants found at endline were more likely to have attended postsecondary schooling, have never been pregnant, and have fewer children. The impact analysis controls for these baseline characteristics and the mode of survey administration. Missing follow-up observations were imputed with baseline values (last observation carried forward), which in this study was no contraceptive use on study entry.

### Effects of Intervention Use

Table 4 presents preliminary evidence of the impact of the investigational intervention on the adoption of contraception. We found that assignment to the treatment group (ie, assignment to receive an encouragement to try the intervention) led to an increase of 12.7 percentage points in the likelihood of contraception use. This is the reduced form estimate (ie, the effect of the invitation—encouragement—on the uptake of contraception). The causal effect of interest, however, is the ratio of the reduced form estimate to the first stage estimate: 41.0 percentage points. This effect is known as LATE, and it represents the average causal effect for women whose use of the intervention was determined only through the random encouragement to try the intervention. In other words, it is *the effect of using the intervention on contraceptive uptake.* The sign of this estimate appears to be positive, but the CI is wide.

Two additional specifications are presented in Multimedia Appendix 3: (1) ordinary least squares estimates produced without the use of last observation carried forward imputation for missing data and (2) probit regression estimates. In the models based on the subset of complete data (1), the estimates and CIs are slightly wider than those presented in Table 4. In the nonlinear specifications (2), the results are consistent with the linear results presented in Table 4.

**Table 3:** Baseline participant characteristics by follow-up status.

Characteristic	Not found (N=62)	Found (N=50)	*P* value^a^
Assigned to treatment, n (%)	27 (44)	29 (58)	.18
Age, mean (SD)	25.0 (5.0)	24.4 (4.6)	.54
Married or in union, n (%)	39 (63)	26 (52)	.33
Christian, n (%)	58 (94)	49 (98)	.50
Luhya tribe, n (%)	47 (76)	39 (78)	.96
Attended postsecondary schooling, n (%)	7 (11)	14 (28)	.045
No schooling, n (%)	2 (3)	0 (0)	.57
Nulligravida, n (%)	15 (24)	21 (42)	.07
Number of children born, mean (SD)	1.8 (1.7)	1.2 (1.3)	.04
Number of desired children, mean (SD)	3.7 (1.4)	3.4 (1.1)	.13
Unmet need for spacing, %	47 (76)	43 (86)	.27
Past use of family planning, n (%)	46 (74)	34 (68)	.61
Number methods known, mean (SD)^b^	9.4 (2.3)	9.8 (2.1)	.31
Not exposed to family planning messages^c^, n (%)	14 (23)	8 (16)	.53

^a^Two-sample *t* tests of mean differences and two-proportions z tests of differences in proportions.

^b^Asked about knowledge of 12 different methods.

^c^Did not hear or see a family planning message on a radio or television or read in a newspaper or magazine in the past few months.

**Table 4:** Impact on contraception adoption (N=122).

Model details^a^	Tried intervention	Adopted contraception	
	First stage regression estimate^b^ (95% CI)	Intent-to-treat estimate^c^ (95% CI)	Instrumental variables estimate^d^ (95% CI)
Assigned to treatment	.31 (0.19 to 0.44)	.13 (−0.01 to 0.26)	N/A^e^
Tried intervention	N/A	N/A	.41 (−0.03 to 0.85)
Mean in control group	.02	.16	N/A

^a^Models include the following controls: an indicator for mode of follow-up survey administration and several baseline characteristics, including age, number of children born, and indicators for having attended postsecondary schooling, past use of family planning, being married or in a union, and nulligravida.

^b^The first stage regression estimate is the coefficient on assignment to treatment from an ordinary least squares regression of intervention use on assignment.

^c^The intent-to-treat estimate is the coefficient on assignment to treatment from an ordinary least squares regression of contraception adoption on assignment.

^d^The instrumental variables estimate is the coefficient on intervention use in a two-stage least squares regression of contraception adoption on assignment and intervention use.

^e^N/A: not applicable.

## Discussion

### Principal Findings

This pilot study demonstrates that the proposed recruitment, encouragement, and data collection procedures are feasible, but some modifications are necessary prior to conducting a full trial. Additionally, analysis of the pilot data suggests that the investigational intervention may have a positive effect on contraceptive uptake among women with an unmet need in Kenya, but a full trial is required to replicate the direction of this effect and more precisely estimate the effect size.

During a recruitment period that lasted 4 weeks, we screened 772 women for eligibility, but only enrolled 14.5% (112/772) in the study. At this rate, it would have taken another week to reach our original target sample size. While this approach was feasible in terms of time and resources, it was inefficient in two ways. First, two-thirds of women who were ineligible to enroll did not meet the basic eligibility criteria such as age, residence, and phone ownership. Screening out these women was not time intensive, but we could have eliminated some work and inconvenience to interested women by more clearly stating the criteria on the market stall signage. Second, 1 out of every 3 ineligible women was ineligible because they did not have an unmet need for family planning. To some extent, this was unavoidable because we did not directly recruit women with an unmet need, but rather embedded checks for eligibility in a short screening available to all women in the eligible age range. In a future trial, it may be advantageous to recruit from other subpopulations in addition to open-air markets to increase the probability that the pool of potential participants will have an unmet need. For instance, recruiting from postsecondary institutions would enable us to reach younger, unmarried women who may be sexually active but not using contraception. Postnatal clinics are another potential venue for recruitment as there is a high unmet need among new mothers in this region.

We used a randomized encouragement design to account for expected two-sided noncompliance with treatment assignment. Women assigned to the treatment group received an invitation via an SMS text message to try the intervention, and 33.9% of these women accepted the invitation, a conversion rate that appears to be consistent with SMS text message marketing conversion rates observed in industry [31]. By comparison, 1.8% of control participants tried the intervention. The encouragement led to a differential rate of intervention uptake of 32.1 percentage points, thereby making causal identification possible using assignment to treatment as an instrument.

The intervention uptake rate is important because incomplete uptake requires an inflation of sample size estimates that are based on fixed parameters for power, alpha, and the desired minimal detectable effect size for traditional randomized controlled trials. Another important consideration for the optimal sample size is attrition. In this study, 44.6% of enrolled participants completed the follow-up survey via an SMS text message or a phone call with a study enumerator. We did not collect detailed tracking information from participants during the recruitment process, so we could only invite participants to complete the survey via an SMS text message. In a future trial, it will be important to have the option to conduct in-person follow-up to reduce study attrition. Other studies that relied solely on SMS text message invitations as we did have encountered similar challenges [13].

A third key consideration for sample size calculations is the minimal detectable effect size. In this study, the instrumental variables estimate of the treatment effect was an increase in the likelihood of contraception uptake of 41.0 percentage points among the treatment group members who tried the intervention. This is an approximate standardized effect size of 1.1; however, this is only a point estimate, and 95% CI is wide. While the results suggest that the intervention effect may be positive, the point estimate is not measured precisely. The effect observed in this study is large relative to other SMS text message interventions for health behavior change [13,32,33], so it will be important to use a more conservative estimate to determine the optimal sample size for the full trial.

### Limitations

The main limitation of this study was attrition. While attrition was not significantly associated with treatment assignment, found and unfound participants at endline differed on a few baseline characteristics. The preliminary impact analysis controls for these differences, but selection bias is a concern. Our reliance on self-reported data, while standard for a trial like this, also has the potential for bias.

As this study was conducted in only one, largely rural county in Kenya, the results may not generalize to urban or international markets. Additionally, the study was conducted at a unique and challenging time. A few days after the end of the recruitment period, Kenyans voted in a national election that was ultimately nullified by the Supreme Court. A second election took place on October 26, 2017, roughly 2 weeks after the treatment group was encouraged to try the intervention. Then in early November, a 5-month national nurse’s strike came to an end, and nurses around the country—including the bulk of the country’s family planning service providers—returned to work. In short, the pilot study was conducted during a period of uncertainty, likely distrust of SMS text message marketing amid heavy political advertising, and a significant decrease in the availability of family planning providers. Given these extenuating circumstances, we attempted to follow-up with participants approximately 4 months after the treatment group was encouraged to try the service rather than 1 month as originally planned.

### Conclusions

This randomized encouragement design and study protocol is feasible but requires modifications to the recruitment, encouragement, and follow-up data collection procedures.

## Appendix

Multimedia Appendix 1 Survey instruments.

Multimedia Appendix 2 Algorithm for determining unmet need.

Multimedia Appendix 3 Supplemental tables.

Multimedia Appendix 4 CONSORT‐EHEALTH checklist (V 1.6.1).

## Abbreviations

DHS: Demographic and Health Survey
LATE: local average treatment effect
SMS: short message service
