# Supplementary material for: Developing a Digital Marketplace for Family Planning: Pilot Randomized Encouragement Trial
Source: J Med Internet Res. 2018 Jul 31;20(7):e10756. doi: 10.2196/10756 (PMC6092593; doi:10.2196/10756)
Supplement: Multimedia Appendix 1 [file jmir_v20i7e10756_app1.pdf]

# Appendix B

recruitment eligibility screen



| name | label::English | label::Swahili | relevant | required | options.english | options.swahili |
|---|---|---|---|---|---|---|
| start | A1. Start Time | A1. Start Time | | yes | | |
| end | A2. End Time | A2. End Time | | yes | | |
| today | A3. Date of Survey | A3. Date of Survey | | yes | | |
| deviceid | A4. Device | A4. Device | | yes | | |
| beginEligibilty1 | | | | | | |
| Ins6 | B1. Enumerator: "I'm going to ask you a few questions to see if you are eligible to complete the survey. Not everyone will be eligible. If you are not eligible or if you choose not to join the study, we will only use several key responses to summarize who screens ineligible. We will not use your information for any other purpose. We will not record your name unless you choose to join the study. Please answer honestly. There are no right or wrong answers." | B1. Enumerator: "Naenda kukuuliza maswali machache kuona kama unafaa kukamilisha utafiti. Siyo kila mtu atafaa kwa utafiti huu. Ikiwa haufai au ikiwa Utachagua/utaamua kutojiunga na utafiti huu, tutatumia tu majibu machache muhimu ili kukamilisha awamu ya asiyefaa. Hatutatumia ujumbe wako kwa madhumuni mengine yoyote. Hatutanakili jina lako kama utachagua kutoshiriki kwenye utafiti. Tafadhali uyajibu kiukweli. Hakuna majibu sawa/ ya kweli ama yasiyo sawa /yasiyo kweli." | | | | |
| New8 | B2. How old are you? | B2. Uko na umri/miaka mingapi? | | yes | #N/A | #N/A |
| New2 | B3. In which Kenyan COUNTY do you reside? | B3. Unaishi kaunti gani hapa kenya? | | yes | Baringo, Bomet, Bungoma, Busia, Elgeyo Marakwet, Embu, Garissa, Homa Bay, Isiolo, Kajiado, Kakamega, Kericho, Kiambu, Kilifi, Kirinyaga, Kisii, Kisumu, Kitui, Kwale, Laikipia, Lamu, Machakos, Makueni, Mandera, Meru, Migori, Marsabit, Mombasa, Muranga, Nairobi, Nakuru, Nandi, Narok, Nyamira, Nyandarua, Nyeri, Samburu, Siaya, Taita Taveta, Tana River, Tharaka Nithi, Trans Nzoia, Turkana, Uasin Gishu, Vihiga, Wajir, West Pokot | Baringo, Bomet, Bungoma, Busia, Elgeyo Marakwet, Embu, Garissa, Homa Bay, Isiolo, Kajiado, Kakamega, Kericho, Kiambu, Kilifi, Kirinyaga, Kisii, Kisumu, Kitui, Kwale, Laikipia, Lamu, Machakos, Makueni, Mandera, Meru, Migori, Marsabit, Mombasa, Muranga, Nairobi, Nakuru, Nandi, Narok, Nyamira, Nyandarua, Nyeri, Samburu, Siaya, Taita Taveta, Tana River, Tharaka Nithi, Trans Nzoia, Turkana, Uasin Gishu, Vihiga, Wajir, West Pokot |
| New3 | B4. In which subcounty within Bungoma county do you reside? | B4. Unaishi kaunti ndogo (sub-kaunti) gani hapa kaunti ya Bungoma? | ${New2} = 'Bungoma' | yes | Bumula, Kanduyi, Sirisia, Kabuchai, Kimilili, Tongaren, Webuye East, Webuye West, Mt. Elgon | Bumula, Kanduyi, Sirisia, Kabuchai, Kimilili, Tongaren, Webuye East, Webuye West, Mt. Elgon |
| New30 | B5. At which market are you taking this survey? | B5. Unafanyia utafiti huu katika soko gani? | | yes | Webuye, Bungoma, Misikhu, Chwele, Kimilili, Lugulu | Webuye, Bungoma, Misikhu, Chwele, Kimilili, Lugulu |
| New9 | B6. Do you own a phone? | B6. Unamiliki/uko na simu? | | yes | Yes, No | Ndio, La |
| New13 | B7. Do you have the phone with you right now? | B7. Uko na simu kwa sasa? | ${New9} = 'Yes' | yes | Yes, No | Ndio, La |
| New14 | B8. Do you share your phone with anyone else? | B8. Huwa unatumia simu yako na mtu mwingine? | ${New13} = 'Yes' | yes | Yes, No | Ndio, La |
| New15 | B9. Are you the primary owner of the phone? | B9. Ikiwa unatumia simu yako na mtu mwingine,wewe ndiye mwenye simu hiyo? | ${New14} = 'Yes' | yes | Yes, No | Ndio, La |
| New12 | B10. Would you be willing to receive follow-up SMS messages or phone calls from the study team in the future? | B10. Ungependa kupokea ujumbe mfupi wa kufuatilia ama kupigiwa simu na timu ya utafiti wakati ujao? | ${New9} = 'Yes' | yes | Yes, No | Ndio, La |
| check1 | if(${New12} = 'Yes' and ${New8} >= 18 and ${New8} <= 35 and ${New2} = 'Bungoma' and ${New9} = 'Yes' and ${New13} = 'Yes' and (${New14} = 'No' or ${New15} = 'Yes') , 1, 0) | | | | | |
| New6 | B13. Enumerator: "Studies often look for different types of people. There was nothing wrong about your answers, however today you are not eligible for this study as designed. Thank you for your time." (0) | B13. Enumerator: "Wakati mwingine utafiti hutafuta/chagua watu tofauti kushiriki.Hakuna kitu mbaya na majibu yako, lakini kwa leo/ wakati huu haufai kwa utafiti huu jinsi ulivyoundwa/ tengenezwa. Asante kwa wakati wako…. " (0) | ${check1}=0 | | | |
| New10 | B14. [Send a SMS to 22384 with the words dghi test +254XXXXXXXXX. Record the result.] | B14. [Send a SMS to 22384 with the words dghi test +254XXXXXXXXX. Record the result.] | ${check1}=1 | yes | Participant phone number already exists, Participant did not receive text, OK to proceed | Participant phone number already exists, Participant did not receive text, OK to proceed |
| New42 | B15. Enumerator: "It appears that your phone number is not compatible with our study at this time. You will not need to complete the rest of the survey. Thank you and have a great day." (0) | B15. Enumerator: "Inaonekana kwamba nambari ya simu yako hailingani na utafiti wetu kwa sasa.Kwa hivo hautakamilisha ule utafiti uliosalia/baki. Asante na kuwa na siku njema." (0) | ${New10}='number_exists' or ${New10}='did_not_receive' | | | |
| passedEligibility1 | if(${check1}=1 and ${New10}='ok', 1, 0) | | | | | |
| endEligibility1 | | | | | | |
| beginEligibilty2 | | | ${passedEligibility1}=1 | | | |
| Ins7 | C1. Enumerator: "Great. Let me ask you a few more questions to determine if you are eligible to complete the survey. If you would not like to answer a particular question, please select the "refuse" option." | C1. Enumerator: "Vyema. Hebu nikuulize maswali machache zaidi, ili kudhibitisha kama unafaa kukamilisha utafiti huu. Ikiwa hautapendelea kujibu swali fulani,tafadhali chagua jibu ya "Kataa"." | | | | |
| DHSR226 | C2. Are you pregnant now? | C2. Wewe ni mjamzito kwa sasa? | | yes | Yes, No, Unsure | Ndio, La, Sina uhakika |
| DHSC303 | C3. Are you currently doing something or using any method to delay or avoid getting pregnant? | C3. Kwa sasa, unatumia mbinu zozote kuchelewa ama kuepuka kupata/kushika mimba? | | yes | Yes, No | Ndio, La |
| DHSC304 | C4. Which method are you using? SELECT ALL THAT APPLY. | C4. Ni mbinu gani unayotumia? CHAGUA ZOTE ZIFAAYO. | ${DHSC303} = 'Yes' | yes | Female Sterilization/TL, Male Sterillization, IUD/ Coil, Injectables, Implants, Pill, Male Condom, Female Condom, Lactational Amenorrhea Method, Safe days/Calendar Method, Withdrawal, Other Modern Method, Other Traditional Method | Kutasisha kike/TL, Kutasisha mume, Koili, Sindano, Vidude za kupanga uzazi, Tembe, Kondomu ya wanaume, Kondomu ya wanawake, Njia ya kupanga uzazi kupitia unyonyeshaji wa mtoto, Mbinu ya kuhesabu masiku/ calenda, Uondoaji, Mbinu zingine za kisasa, Mbinu zingine za mila |
| DHSC313 | C5. Have you ever used anything or tried in any way to delay or avoid getting pregnant? | C5. Umewahi tumia kitu chochote ama kujaribu kwa njia zozote kuchelewa ama kuepuka kupata/kushika mimba? | ${DHSC303} = 'No' | yes | Yes, No, Refuse | Ndio, La, Kataa |
| passedEligibility2 | if(${DHSR226} = 'Yes' or ${DHSC303} = 'Yes', 0, 1) | | | | | |

| | | | | | | |
|---|---|---|---|---|---|---|
| New6 | C7. Enumerator: "Studies often look for different types of people. There was nothing wrong about your answers, however today you are not eligible for this study as designed. Thank you for your time." (0) | C7. Enumerator: "Wakati mwingine utafiti hutafuta/chagua watu tofauti kushiriki.Hakuna kitu mbaya na majibu yako, lakini kwa leo/ wakati huu haufai kwa utafiti huu jinsi ulivyoundwa/ tengenezwa. Asante kwa wakati wako.... " (0) | ${passedEligibility2} = 0 | | | |
| endEligibility2 | | | | | | |
| BeginEligibilty3 | | | ${passedEligibility2}=1 | | | |
| Ins1 | D1. [Use the following questions to show how the tablet works. Explain how to swipe to continue and to read questions carefully.] | D1. [Use the following questions to show how the tablet works. Explain how to swipe to continue and to read questions carefully.] | | | | |
| New32 | D2. What is the color of the sun? | D2. Jua ni rangi gani? | | yes | Yellow, Black, Grey, Blue, Green | Manjano , Nyeusi , Kijivu , Zamawati , Kijani kibichi |
| New33 | D3. Which of the following are foods? Select all that apply. | D3. Ni gani kati ya hizi ni chakula? chagua zote zifaazo. | | yes | Ugali, Chapati, House, Sukuma, Matatu | Ugali, Chapati, Nyumba, Sukuma, Matatu |
| New22 | D4. Enumerator: "We would like to ask a few practice questions before beginning the survey." [Hand tablet to participant] | D4. Enumerator: "Tungependa kukuuliza maswali chache ya mazoezi kabla ya kuanza utafiti." [Hand tablet to participant] | | | | |
| Ins2 | D5. [Let participant go through the next three practice questions on their own one time. Do not help them. Follow future prompts.] | D5. [Let participant go through the next three practice questions on their own one time. Do not help them. Follow future prompts.] | | | | |
| New23a | D6. Which COUNTRY are you in? | D6. Uko nchi gani? | | yes | Uganda, South Africa, Pluto, Tanzania, Kenya | Uganda, Afrika kusini, Pluto, Tanzania , Kenya |
| New24a | D7. Which of the following are animals? Select all that apply. | D7. Gani kati ya hawa ni wanyama? Chagua yote yafaayo. | | yes | Giraffe , Sheep, Car, Goat, Book, Computer | Twiga, Kondoo, Gari, Mbuzi, Kitabu , Tarakalishi |
| New25a | D8. What is the current year? (YYYY) | D8. Huu ni mwaka gani? (YYYY) | | yes | #N/A | #N/A |
| New26a | D9. Thank you for completing the practice survey. Please take the tablet to the enumerator. | D9. Asante kwa kukamilisha utafiti wa mazoezi. Tafadhali rudisha kibao simu kwa mtafiti. | | yes | | |
| Test1 | if(not(${New23a} = 'Kenya' and selected(${New24a}, 'Giraffe') and ${New25a} = 2017 and selected(${New24a}, 'Sheep') and selected(${New24a}, 'Goat') and not(selected(${New24a}, 'Car') or selected (${New24a}, 'Computer') or selected (${New24a}, 'Book'))), 0, 1) | | | | | |
| New27a | D11. [Repeat tablet instructions and ask the participant to try again. Do not help them.] | D11. [Repeat tablet instructions and ask the participant to try again. Do not help them.] | ${Test1}=0 | | | |
| New23b | D12. Which COUNTRY are you in? | D12. Uko nchi gani? | ${Test1}=0 | yes | Uganda, South Africa, Pluto, Tanzania, Kenya | Uganda, Afrika kusini, Pluto, Tanzania , Kenya |
| New24b | D13. Which of the following are animals? Select all that apply. | D13. Gani kati ya hawa ni wanyama? Chagua yote yafaayo. | ${Test1}=0 | yes | Giraffe , Sheep, Car, Goat, Book, Computer | Twiga, Kondoo, Gari, Mbuzi, Kitabu , Tarakalishi |
| New25b | D14. What is the current year? (YYYY) | D14. Huu ni mwaka gani? (YYYY) | ${Test1}=0 | yes | | |
| New26b | D15. Thank you for completing the practice survey. Please take the tablet to the enumerator. | D15. Asante kwa kukamilisha utafiti wa mazoezi. Tafadhali rudisha kibao simu kwa mtafiti. | ${Test1}=0 | yes | | |
| Test2 | if(not(${New23b} = 'Kenya' and selected(${New24b}, 'Giraffe') and ${New25b} = 2017 and selected(${New24b}, 'Sheep') and selected(${New24b}, 'Goat') and not(selected(${New24b}, 'Car') or selected (${New24b}, 'Computer') or selected (${New24b}, 'Book'))), 0, 1) | | ${Test1}=0 | | | |
| New27b | D17. [Repeat tablet instructions and ask the participant to try again. Do not help them.] | D17. [Repeat tablet instructions and ask the participant to try again. Do not help them.] | ${Test2}=0 | | | |
| New23c | D18. Which COUNTRY are you in? | D18. Uko nchi gani? | ${Test2}=0 | yes | Uganda, South Africa, Pluto, Tanzania, Kenya | Uganda, Afrika kusini, Pluto, Tanzania , Kenya |
| New24c | D19. Which of the following are animals? Select all that apply. | D19. Gani kati ya hawa ni wanyama? Chagua yote yafaayo. | ${Test2}=0 | yes | Giraffe , Sheep, Car, Goat, Book, Computer | Twiga, Kondoo, Gari, Mbuzi, Kitabu , Tarakalishi |
| New25c | D20. What is the current year? (YYYY) | D20. Huu ni mwaka gani? (YYYY) | ${Test2}=0 | yes | | |
| New26c | D21. Thank you for completing the practice survey. Please take the tablet to the enumerator. | D21. Asante kwa kukamilisha utafiti wa mazoezi. Tafadhali rudisha kibao simu kwa mtafiti. | ${Test2}=0 | yes | | |
| Test3 | if(not(${New23c} = 'Kenya' and selected(${New24c}, 'Giraffe') and ${New25c} = 2017 and selected(${New24c}, 'Sheep') and selected(${New24c}, 'Goat') and not(selected(${New24c}, 'Car') or selected (${New24c}, 'Computer') or selected (${New24c}, 'Book'))), 0, 1) | | ${Test1}=0 | | | |
| Ins3 | D24. Enumerators: "These tablets can be difficult to use. It looks like you're struggling a bit, so this may not be a fun task. You will not need to continue the survey. Thank you for your time. Have a nice day." (0) | D24. Enumerator: "Hivi vibao simu vinaweza kuwa ngumu kutumia. Inaonekana kana kwamba unang'ang'ana kiasi, kwa hivo huenda isiwe kazi ya kukufurahisha.<br>Kwa hivo hautakamilisha utafiti. Asante kwa muda wako. Kuwa na siku njema." (0) | ${Test1}=0 and ${Test2}=0 and ${Test3}=0 | | | |
| passedEligibility3 | if(${Test1}=1 or ${Test2}=1 or ${Test3}=1, 1, 0) | | | | | |
| endEligibility3 | | | | | | |
| BeginUnmetNeed | | | ${passedEligibility3}=1 | | | |
| New41 | E1. Enumerator: "This survey will ask you some personal questions about family planning. You are welcome to take the survey on your own and read and/or listen to the questions. Or if you'd like, I can ask you the questions and you can tell me your answers. It's up to you. Would you like to do this on your own, or would you like help?" | E1. Enumerator: "Utafiti huu utakuuliza maswali ya kibinafsi kuhusu upangaji uzazi. Unakaribishwa kufanya utafiti huu wewe mwenyewe/wewe binafsi usome, na usikilize maswali, ama ikiwa utapenda nikuulize maswali na uniambie majibu, uamuzi ni wako. Je, ungependa kujifanyia/ kufanya wewe mwenyewe ama ungependa usaidiwe?" | | yes | By Herself, By the Enumerator | By Herself, By the enumerator |
| Ins5 | E2. Enumerator: "Great. If you need help at any point, just ask me." [Give the tablet to the participant] | E2. Enumerator: "Vyema, kama utahitaji usaidizi wakati wowote, niulize tu." [Give the tablet to the participant] | ${New41}='self' | | | |

| | | | | | | |
|---|---|---|---|---|---|---|
| New16 | E3. Welcome and thank you for agreeing to take the Women's Health survey. Your responses to these questions will be confidential. This survey should take you about 15 to 30 minutes to complete. When you are done, we will send you airtime as a thank you for your time. Please swipe right to continue. | E3. Karibu na asante sana kwa kukubali kushiriki kwa utafiti wa Afya ya Wanawake. Majibu yako kwa haya maswali itakuwa ya siri. Utafiti unawezachukua muda wa dakika 15 hadi 30. Utakapomaliza, tutakutumia kadi ya simu kama pongezi kwa muda wako. Unaweza endelea. | | | | |
| New29 | E4. Please enter the last 4 digits of your phone number. | E4. Tafadhali weka nambari 4 za mwisho za simu yako. | | yes | | |
| New4 | E5. You will now complete a series of questions regarding your family. | E5. Sasa utakamilisha mfulullizo wa maswali kuhusu familia yako. | | | | |
| DHSR201 | E6. Have you ever given birth? | E6. Umewahi jifungua? | | yes | Yes, No | Ndio, La |
| DHSR202 | E7. Do you have any sons or daughters to whom you have given birth who are now living with you? | E7. Unao watoto wavulana au wasichana wenye umewazaa na unaishi nao? | ${DHSR201} = 'Yes' | yes | Yes, No | Ndio, La |
| DHSR203A | E8. How many sons live with you? IF NONE, RECORD '0'. | E8. Ni watoto wavulana wangapi wanaishi na wewe? KAMA HAKUNA REKODI '0'. | ${DHSR202} = 'Yes' | yes | | |
| DHSR203B | E9. How many daughters live with you? IF NONE, RECORD '0'. | E9. Ni watoto wasichana wangapi wanaishi na wewe? KAMA HAKUNA REKODI '0'. | ${DHSR202} = 'Yes' | yes | | |
| DHSR204 | E10. Do you have any sons or daughters to whom you have given birth who are alive but do not live with you? | E10. Unao wavulana au wasichana wenye ulizaa na wako hai lakini hawaishi na wewe? | ${DHSR201} = 'Yes' | yes | Yes, No | Ndio, La |
| DHSR205A | E11. How many sons are alive but do not live with you? IF NONE, RECORD '0'. | E11. Ni wavulana wangapi wako hai na hawaishi na wewe? KAMA HAKUNA,REKODI '0'. | ${DHSR204} = 'Yes' | yes | | |
| DHSR205B | E12. How many daughters are alive but do not live with you? IF NONE, RECORD '0' | E12. Ni wasichana wangapi wako hai na hawaishi na wewe? KAMA HAKUNA, REKODI '0'. | ${DHSR204} = 'Yes' | yes | | |
| DHSR206A | E13. Have you ever given birth to a boy or girl who was born alive but later died? | E13. Ushawai jifungua mtoto mvulana au msichana, mwenye alizaliwa akiwa hai na baadaye akafa/akaaga dunia? | | yes | Yes, No | Ndio, La |
| DHSR206B | E14. Have you ever given birth to a baby who cried or showed signs of life but did not survive? | E14. Ushawai jifungua mtoto mwenye alilia au aliyeonyesha dalili za uhai lakini hakuishi? | ${DHSR206A} = 'No' | yes | Yes, No | Ndio, La |
| DHSR207A | E15. How many boys have died? IF NONE, RECORD '0'. | E15. Ni wavulana wangapi washawai aga dunia/kufa? KAMA HAKUNA, REKODI '0'. | ${DHSR206A} = 'Yes' or ${DHSR206B} = 'Yes' | yes | | |
| DHSR207B | E16. How many girls have died? IF NONE, RECORD '0'. | E16. Ni wasichana wangapi washawai aga dunia/kufa? KAMA HAKUNA, REKODI '0'. | ${DHSR206A} = 'Yes' or ${DHSR206B} = 'Yes' | yes | | |
| DHSR209 | if(${DHSR203A}>=0,${DHSR203A},0)+if(${DHSR203B}>=0,${DHSR203B},0)+if(${DHSR205A}>=0,${DHSR205A},0)+if(${DHSR205B}>=0,${DHSR205B},0)+if(${DHSR207A}>=0,${DHSR207A},0)+if(${DHSR207B}>=0,${DHSR207B},0) | | | yes | | |
| DHSR209A | E18. Just to make sure that I have this right: you have had in TOTAL ${DHSR209} births during your life. Is that correct? | E18. Ili kudhibitisha nimekupata vizuri: kwa jumla, umewahi jifungua mara ${DHSR209} kwa maisha yako. Je hio ni sahihi? | | yes | Yes, No | Ndio, La |
| DHSR209B | E19. In TOTAL how many births have you had during your life? Please enter the correct number. | E19. Kwa JUMLA umewahi zaa mara ngapi kwa maisha yako? | ${DHSR209A} = 'No' | yes | | |
| DHSFP712A | E20. If you could choose or if you could have chosen exactly the number of children to have in your whole life how many would that be? | E20. Kama ungechagua idadi ya watoto ungetaka kwa maisha yako yote, wangekuwa wangapi? | | yes | | |
| New19 | E21. When was your last birth? | E21. Ulijifungua lini mara ya mwisho? | ${DHSR201} = 'Yes' | yes | Less Than 2 Years Ago, Within the Last 2 to 5 Years, More Than 5 Years Ago, Don't Know | Chini ya miaka 2 iliyopita, Kati ya miaka 2 hadi 5 iliyopita, Zaidi ya miaka 5 iliyopita, Sijui |
| DHSR228 | E22. When you got pregnant, did you want to get pregnant at that time? | E22. Uliposhika/pata mimba, ulikuwa unataka uwe mjamzito wakati huo? | ${DHSR201} = 'Yes' | yes | Yes, No | Ndio, La |
| DHSR229 | E23. If you did not want to get pregnant at that time, did you want to have a baby later on or did you not want any(more) children? | E23. Ikiwa hukutaka kupata mimba wakati huo, ungependa kupata mtoto baadaye au hukuwa unataka mtoto/watoto wengine? | ${DHSR228} = 'No' | yes | Later, No More | Baadaye, Singependa watoto wengine |
| DHSR238 | E25. When did your last menstrual period start? | E25. Hedhi/ damu ya mwezi yako ya mwisho ilianza lini? | | yes | Less than 6 months ago, Between 6 months and 2 years ago, Between 2 years and 5 years ago, More than 5 years ago, In Menopause/Have Had A Hysterectomy , Never Menstruated | Chini ya miezi 6 iliyopita, Kati ya miezi 6 na miaka 2 iliyopita, Kati ya miaka 2 na miaka 5 iliyopita, Zaidi ya miaka 5 iliyopita, Nimemaliza kuona hedhi/damu ya mwezi/ nimetoa nyumba ya mtoto, Sijawahi ona hedhi/ damu ya mwezi |
| group2 | if(${New19}='Less_Than_2_Years_Ago' and (${DHSR238}='Between_24_and_60_months' or ${DHSR238}='More_than_60_months'), 1, 0) | | | | | |
| group2Need | if(${DHSR228}='Yes', 'no_unmet_need', 'unmet_need') | | ${group2}=1 | | | |
| group2UnmetType | if(${DHSR229}='Later', 'spacing', 'limiting') | | ${group2Need}='unmet_need' | | | |
| DHSFP704 | E29. Would you like to have (a/another) child, or would you prefer not to have any (more) children? | E29. Ungependa kupata mtoto (mwingine) ama ungependelea kutokuwa na mtoto/watoto wengine tena? | | yes | Have (A/Another) Child, No More Children, I Can't Get Pregnant, Undecided/Don't Know | Kuwa na mtoto/kupata mtoto mwingine, Kutopata watoto wengine, Siwezi shika mimba, Sijui |
| DHSFP705 | E30. How long would you like to wait from now before the birth of (a/another) child? | E30. Unatarajia kungoja kwa muda gani kutoka sasa kabla kujifungua mtoto wa kwanza/mwingine? | ${DHSFP704} = 'Have_AAnother_Child' | yes | Soon/Now , Not now, but within 2 years, More than 2 years from now, I Can't Get Pregnant, After Marriage, Other, Don't Know | Hivi karibuni/saa hii, Si kwa sasa, lakini kwa muda wa miaka miwili, Zaidi ya miaka miwili kuanzia sasa , Siwezi shika mimba, Baada ya ndoa, Ingine, Sijui |
| DHSMSA601 | E31. Are you currently married or living together with a man as if married? | E31. Kwa sasa, umeolewa ama unaishi na mwanumume kana kwamba umeolewa? | | yes | Yes, Currently Married, Yes, Living With A Man, No, Not In Union | Ndio, nimeolewa kwa sasa, Ndio, naishi na mwanamume, La, siko kwa uhusiano |
| DHSMSA602 | E32. Have you ever been married or lived with a man as if married? | E32. Umewahiolewa ama kuishi na mwanamume kana kwamba umeolewa? | ${DHSMSA601} = 'No,_Not_In_Union' | yes | Yes, Formerly Married, Yes, Lived With A Man, No | Ndio, niliolewa zamani, Ndio, niliiishi na mwanamume, La |
| DHSMSA603 | E33. What is your marital status now: are you widowed, divorced, or separated? | E33. Hali yako ya ndoa kwa sasa iko vipi: wewe ni mjane, umetalakiwa ama mlitengana? | ${DHSMSA602} = 'Yes' | yes | Widowed, Divorced, Separated | Mjane, Talaka, Tengana |

| | | | | | | |
|---|---|---|---|---|---|---|
| DHSFP718 | E34. Would you say that using contraception is mainly your decision, mainly your husband's/partner's decision, or did you both decide together? | E34. Ungesema kuwa upangaji uzazi ni uamuzi wako wewe mwenyewe, uamuzi wa mume/mpenzi wako ama mliamua nyinyi nyote kwa pamoja? | ${DHSMSA601}='Yes,_Currently_Married' or ${DHSMSA601}='Yes,_Living_With_A_Man' | yes | Mainly Mine, Mainly My Husband's/Partner's, Joint Decision, Other | Mimi mwenyewe, Mume/mpenzi wangu hasa, Uaumuzi wa pamoja, Mengine |
| DHSFP718A | E35. Specify Other: | E35. Taja mengine: | ${DHSFP718}='Other' | | | |
| DHSFP720 | E36. Does your husband/partner want the same number of children that you want, or does he want more or fewer than you want? | E36. Je, mume/mpenzi wako anataka idadi sawa ya watoto unaotaka kupata, au anataka wengi au wachache kuliko unaotaka? | ${DHSMSA601}='Yes,_Currently_Married' or ${DHSMSA601}='Yes,_Living_With_A_Man' | yes | Same Number, More Children, Fewer Children, Don't Know | Idadi sawa, Watoto wengi, Watoto wachache, Sijui |
| New21 | E37. Did you start living with your husband/partner 5 or more years ago? | E37. Ulianza kuishi na mume/mpenzi wako miaka tano iliyopita au zaidi ? | ${DHSMSA601}='Yes,_Currently_Married' or ${DHSMSA601}='Yes,_Living_With_A_Man' | yes | Yes, No | Ndio, La |
| DHSFP716A | E40. Do you think that your husband/partner approves or disapproves of couples using a method to avoid pregnancy? | E40. Je, unadhani kuwa mume/mpenzi wako anakubali au hakubali wanandoa kutumia mbinu kuzuia kupata/kushika mimba? | ${DHSMSA601}='Yes,_Currently_Married' or ${DHSMSA601}='Yes,_Living_With_A_Man' | yes | Approves, Disapproves, Don't Know | Anakubali, Hakubali, Sijui |
| DHSFP716B | E41. How often have you talked to your husband/partner about family planning in the past year? | E41. Ni mara ngapi umeongea na mume/mpenzi wako juu ya upangaji uzazi kwa muda wa mwaka moja uliopita? | ${DHSMSA601}='Yes,_Currently_Married' or ${DHSMSA601}='Yes,_Living_With_A_Man' | yes | Never, Once or Twice, More Often | Sijawahi/kamwe, Mara moja ama mara mbili, Kila mara |
| DHSMSA613 | E42. Do you know how old you were when you had sexual intercourse for the very first time? | E42. Je, unajua umri uliyokuwa nao uliposhiriki ngono kwa mara ya kwanza? | | yes | Yes, No, I Have Never Had Sexual Intercourse, My First Time Was When I Started Living With My (First) Husband/Partner | Ndio, La, Sijawai fanya ngono, Mara yangu ya kwanza ni wakati ambayo nilianza kuishi na mume/mpenzi wangu wa kwanza |
| DHSMSA613A | E43. How old you were when you had sexual intercourse for the very first time? | E43. Ulikwa na miaka ngapi ulipo fanya ngono mara ya kwanza? | ${DHSMSA613} = 'Yes' | yes | | |
| DHSMSA615 | E43. When was the last time you had sexual intercourse? | E43. Ulishiriki ngono Mara ya mwisho lini? | ${DHSMSA601}='No,_Not_In_Union' | yes | I Have Never Had Sexual Intercourse | Sijawahi fanya ngono |
| group3 | if(((${New21}='Yes' and ${DHSC313}="No" and (${DHSR201}='No' or ${New19}='More_Than_5_Years_Ago')) or ${DHSFP704}='I_Cant_Get_Pregnant' or ${DHSR238}='menopause_hysterectomy' or ${DHSR238}='More_than_60_months') and ${group2}!=1, 1, 0) | | | | | |
| group4 | if(${group3}!=1, 1, 0) | | | | | |
| group4Need | if(${DHSFP705}='Soon/Now' or ${DHSFP705}='within_2_years', 'no_unmet_need', 'unmet_need') | | ${group4}=1 | | | |
| group4UnmetType | if(${DHSFP704}='No_More/None', 'limiting', 'spacing') | | ${group4Need}='unmet_need' | | | |
| singleActive | if(${DHSMSA615}='within_1_month' or ${DHSMSA615}='between_1_and_3_months' or ${DHSMSA615}='between_4_and_6_months', 1, 0) | | ${DHSMSA601}='No,_Not_In_Union' | | | |
| unmetNeed | if((${group2Need}='unmet_need' or ${group4Need}='unmet_need') and ${singleActive}!=0, 1, 0) | | | | | |
| New7A | E50. Thank you for completing the survey. Please return the tablet to the enumerator. (00) | E50. Asante kwa kukamilisha utafiti. Tafadhali rudisha kibao simu kwa mtafiti. (00). | ${unmetNeed}=0 | yes | | |
| New45 | E51. Enumerator: "Studies often look for different types of people. There was nothing wrong about your answers, however today you are not eligible for this study as designed. Thank you for your time. We will deliver you airtime by sending a text message to your phone." | E51. Enumerator: "Wakati mwingine utafiti hutafuta/chagua watu tofauti kushiriki. Hakuna kitu mbaya na majibu yako, lakini kwa leo/ wakati huu haufai kwa utafiti huu jinsi ulivyoundwa/ tengenezwa. Asante kwa muda wako. Tutakufikishia/kupatia kadi ya simu kwa kutuma ujumbe mfupi kwenye simu yako." | ${unmetNeed}=0 | | | |
| New47a | E52. Did the participant answer the survey questions by listening to the enumerator administer the questions or answering on her own? | E52. Did the participant answer the survey questions by listening to the enumerator administer the questions or answering on her own? | ${unmetNeed}=0 | yes | | |
| New44 | E53. [Text 22384: dghi thanks n e +254XXXXXXXXX.] | E53. [Text 22384: dghi thanks n e +254XXXXXXXXX.] | ${unmetNeed}=0 | | | |
| endUnmetNeed | | | | | | |
| BeginFP | | | ${unmetNeed}=1 | | | |
| DHSC | F1. Now I would like to talk about family planning - the various ways and methods that a couple can use to delay or avoid a pregnancy. | F1. Sasa ningependa tuongee juu ya upangaji uzazi-njia mbalimbali na mbinu ambazo watu wanawezatumia kuchelewa ama kuepuka mimba. | | | | |
| DHSC30101 | F2. Have you ever heard of female sterilization/TL? Women can have an operation to avoid having any more children. | F2. Umewahi sikia kutasisha ya wanawake/TL? Wanawake wanaweza fanyiwa operesheni ili kuepuka kupata watoto wengine. | | yes | Yes, No, Refuse | Ndio, La, Kataa |
| DHSC30102 | F3. Have you ever heard of male sterilization? Men can have an operation to avoid having any more children. | F3. Umewahi sikia kutasisha ya wanaume? Wanaume wanaweza fanyiwa operesheni ili kuepuka kupata watoto wengine. | | yes | Yes, No, Refuse | Ndio, La, Kataa |
| DHSC30103 | F4. Have you ever heard of an IUD/ coil? Women can have a loop or coil placed inside them by a doctor or nurse. | F4. Umewahi sikia kuhusu koili? Wanawake wanaweza wekewa tanzi au songo/koili na daktari ama muuguzi. | | yes | Yes, No, Refuse | Ndio, La, Kataa |
| DHSC30104 | F5. Have you ever heard of injectables? Women can have an injection by a health provider that stops them from becoming pregnant for one or more months. | F5. Umewahi sikia kuhusu sindano? Wanawake wanaweza dungwa sindano na mhudumu wa afya, ili iwazuie kupata mimba kwa muda wa mwezi moja au miezi kadhaa. | | yes | Yes, No, Refuse | Ndio, La, Kataa |
| DHSC30105 | F6. Have you ever heard of implants? Women can have one or more small rods placed in their upper arm by a doctor or nurse which can prevent pregnancy for one or more years. | F6. Umewahi kusikia kuhusu vidude vya kupanga uzazi? Wanawake wanaweza wekewa kijiti moja au nyingi kwa mkono na daktari au muuguzi ili kuzuia kupata/kushika mimba kwa muda wa mwaka moja au miaka nyingi. | | yes | Yes, No, Refuse | Ndio, La, Kataa |
| DHSC30106 | F7. Have you ever heard of the pill? Women can take a pill every day to avoid becoming pregnant. | F7. Umewahi sikia kuhusu donge/ tembe? Wanawake wanaweza meza donge ili waepuke kupata mimba. | | yes | Yes, No, Refuse | Ndio, La, Kataa |
| DHSC30107 | F8. Have you ever heard of a male condom? Men can put a rubber sheath on their penis before sexual intercourse. | F8. Umewahi sikia kuhusu mpira ya kondomu ya wanaume? Wanaume wanaweza weka mpira kwa sehemu yao ya uzazi kabla ya kufanya ngono. | | yes | Yes, No, Refuse | Ndio, La, Kataa |

| | | | | | | |
|---|---|---|---|---|---|---|
| DHSC30108 | F9. Have you ever heard of a female condom? Women can place a sheath in their vagina before sexual intercourse. | F9. Umewahi sikia kuhusu mpira ya kondomu ya wanawake? Wanawake wanaweza weka mpira kwa sehemu yao ya uzazi kabla ya kufanya ngono. | | yes | Yes, No, Refuse | Ndio, La, Kataa |
| DHSC30109 | F10. Have you ever heard of the Lactation Amenorrhea Method (LAM)? If a woman's menstrual period has not returned in the first 6 months after her baby is born, she can avoid pregnancy by breastfeeding frequently, day and night. | F10. Umewahi sikia kuhusu njia ya kunyonyesha mtoto kama njia ya upangaji uzazi? Kama hedhi/ damu ya mwezi ya mwanamke haijarudi kwa miezi sita ya kwanza baada ya mtoto kuzaliwa, anaweza zuia kupata mimba kwa kunyonyesha mara kwa mara, mchana na usiku. | | yes | Yes, No, Refuse | Ndio, La, Kataa |
| DHSC30110 | F11. Have you ever heard of the safe days/ calendar method? To avoid pregnancy, women do not have sexual intercourse on the days of the month they think they can get pregnant. | F11. Umewahi sikia mbinu ya kuhesabu masiku/ calenda? Ili kuepuka kupata mimba, wanawake hawashiriki kwa ngono siku zenye wanafikiria wanaweza shika/pata mimba. | | yes | Yes, No, Refuse | Ndio, La, Kataa |
| DHSC30111 | F12. Have you ever heard of withdrawal? Men can be careful and pull out before climax. | F12. Umewahi sikia kuhusu uondoaji/ kumwaga inje? Wanaume wanaweza kuwa makini waondoe uume yao kabla ya kufika kilele. | | yes | Yes, No, Refuse | Ndio, La, Kataa |
| DHSC30112 | F13. Have you ever heard of emergency contraception? As an emergency measure, within three days after they have unprotected sexual intercourse, women can take special pills to prevent pregnancy. | F13. Ushawahi sikia kuhusu uzazi wa mpango ya dharura? Kama hatua ya dharura, kati ya siku tatu baada yao kufanya ngono bila kinga , wanawake wanaweza meza donge/ tembe maalum ili kuzuia mimba. | | yes | Yes, No, Refuse | Ndio, La, Kataa |
| DHSC30113A | F14. Have you ever heard of any other ways or methods that women or men can use to avoid pregnancy? | F14. Umewahi sikia kuhusu njia zozote ama mbinu zozote zingine ambazo wanawake au wanaume hutumia kuepuka mimba? | | yes | Yes, No | Ndio, La |
| DHSC30113B | F15. Specify: | F15. Taja: | ${DHSC30113A}='Yes' | | | |
| DHSFP714 | F16. In the last few months have you heard about family planning on the radio? | F16. Kwa miezi michache iliyopita, umewahi sikia kuhusu upangaji uzazi kwa redio.? | | yes | Yes, No, Refuse | Ndio, La, Kataa |
| DHSFP714A | F17. In the last few months have you seen anything about family planning on the television? | F17. Kwa miezi michache iliyopita, umewahi tazama kitu chochote kuhusu upangaji uzazi kwa televisheni? | | yes | Yes, No, Refuse | Ndio, La, Kataa |
| DHSFP714B | F18. In the last few months have you read about family planning in a newspaper or magazine? | F18. Kwa miezi michache iliyopita, umewahi soma kuhusu upangaji uzazi kwa gazeti? | | yes | Yes, No, Refuse | Ndio, La, Kataa |
| DHSFP715A | F19. In the last 12 months have you heard about family planning at public forums such as Barazas or public gatherings? | F19. Kwa muda wa miezi 12 iliyopita, umewahi skia kuhusu upangaji uzazi katika mikutano ya umma, kama baraza au chama | | yes | Yes, No, Refuse | Ndio, La, Kataa |
| DHSFP715B | F20. In the last 12 months have you seen family planning informational material such as posters, brochures, or stickers? | F20. Kwa muda wa miezi 12 iliyopita, umewahi ona habari kuhusu upangaji uzazi kwenye vifaa vya maelezo, kama mkao,vipeperushi au kibandiko | | yes | Yes, No, Refuse | Ndio, La, Kataa |
| DHSFP715C | F21. In the last 12 months have you been visited by a health worker or health professional to discuss family planning issues? | F21. Kwa muda wa miezi 12 iliyopita, ulitembelewa na mhudumu wa afya au mtaalamu wa kiafya kujadiliana kuhusu upangaji uzazi? | | yes | Yes, No, Refuse | Ndio, La, Kataa |
| DHSFP715D | F22. In the last 12 months have you received family planning messages through social media platforms, such as Facebook or twitter? | F22. Kwa muda wa miezi 12 iliyopita, umepokea ujumbe kuhusu upangaji uzazi kupitia mtandao wa kijamii (social media), kama Facebook au Twitter? | | yes | Yes, No, Refuse | Ndio, La, Kataa |
| DHSFP715E | F23. In the last 12 months have you received family planning messages through a mobile phone via text or email? | F23. Kwa muda wa miezi 12 iliyopita, umepokea ujumbe kuhusu upangaji uzazi kupitia simu ya rununu au email? | | yes | Yes, No, Refuse | Ndio, La, Kataa |
| DHSFP715F | F24. In the last 12 months have you heard political / religious / community leaders talk favorably about family planning? | F24. Kwa muda wa miezi 12 iliyopita, umesikia viongozi wa jamii (viongozi wa siasa, dini,jamii) wakizungumza vizuri/wakipendekeza kuhusu upangaji uzazi? | | yes | Yes, No, Refuse | Ndio, La, Kataa |
| endFP | | | | | | |
| BeginHH | | | ${unmetNeed}=1 | | | |
| New5 | G1. Thank you for completing the family planning portion of the survey. You will now be asked some questions about your household and background. | G1. Asante kwa kukamilisha sehemu ya utafiti ya upangaji uzazi. Sasa utaulizwa maswali kuhusu nyumba yako na ulikotoka. | | | | |
| DHSHC102 | G2. What is the main source of drinking water for members of your household? | G2. Maji yenye huwa mnatumia nyumbani hutoka wapi? | | yes | Piped into Dwelling, Piped to Yard/Plot, Public Tap/Standpipe, Tube Well or Borehole, Protected Well, Unprotected Well, Protected Spring, Unprotected Spring, Rainwater, Tanker Truck, Cart with Small Tank, Lake/Pond/Stream/Canal/Irrigation Channel, Bottled Water, Other | Imesambazwa na bombu la maji hadi kwa makao, Imesambazwa na bombu la maji hadi kwa shamba/yadi, Mfereji wa umma, Kisima, Kisima kilicholindwa, Kisima kisicholindwa, Chemichemi iliyolindwa, Chemichemi isiyolindwa, Maji ya mvua, Lori ya maji, Mikokoteni ya maji, Ziwa/bwawa/mkondo/mfereji/kilimo cha umwagiliaji, Maji ya chupa, Mengine |
| DHSHC102A | G3. Specify Other: | G3. Taja hayo mengine: | ${DHSHC102} = 'Other' | | | |
| DHSHC103 | G4. Where is that water source located? | G4. Chanzo cha maji hayo kiko wapi? | | yes | In own Dwelling, In own Yard/Plot, Elsewhere | Kwa nyumba yako, Kwa shamba lako, Mahali pengine |
| DHSHC104A | G5. Do you know how long it takes to go there, get water, and come back? | G5. Je unajua muda wenye unaweza chukua kufika hapo, uteke maji, na urudi? | ${DHSHC103} = 'Elsewhere' | yes | Yes, No | Ndio, La |
| DHSHC104B | G6. How many minutes does it take you to go there, get water, and come back? | G6. Huwa inakuchukua dakika ngapi kufika pale, kuchota maji na kurudi? | ${DHSHC104A} = 'Yes' | yes | | |
| DHSHC105 | G7. Do you do anything to the water to make it safer to drink? | G7. Kuna kitu chochote unafanyia maji ili ikuwe salama kwa kunywa? | | yes | Yes, No, Don't Know, Refuse | Ndio, La, Sijui, Kataa |
| DHSHC106 | G8. What do you usually do to make the water safer to drink? Anything Else? Select all that apply. | G8. Kwa kawaida wewe hufanya nini ili kuweka maji yako yawe salama kwa kunywa? Kitu kingine? Chagua yote yafaayo. | ${DHSHC105} = 'Yes' | yes | Boil, Add Bleach/Chlorine, Strain Through a Cloth, Use Water Filter (Ceramic/Sand/Composite/Etc.), Solar Disinfection, Let it Stand and Settle, Cover the Water Container, Other , Don't Know | Chemsha, Weka klorini kwa maji, Chunga kupitia nguo, Tumia chujio ya maji (kauri,mchanga, na kadhalika), Kusafisha maji kutumia nguvu ya jua/sola, Kuiacha itulie, Funika chombo cha maji, Mengine, Sijui |
| DHSHC106A | G9. Specify Other: | G9. Taja mengine: | ${DHSHC106} = 'Other' | | | |

| | | | | | | |
|---|---|---|---|---|---|---|
| DHSHC107 | G10. What kind of toilet facility do members of your household usually use? | G10. Ni aina gani ya choo wenye nyumba hii hutumia mara kwa mara? | | yes | Flush to Piped Sewer System, Flush to Septic Tank, Flush to Pit Latrine, Flush to Somewhere Else, Flush, Don't Know Where, Ventilated Improved Pit Latrine, Pit Latrine With Slab, Pit Latrine Without Slab/Open Pit, Composting Toilet, Bucket Toilet, No Facility/Bush/Field, Other | Mwaga/flush kupitia mfumo wa mfereji wa maji machafu, Mwaga/flush kwa tanki bora ya maji chafu/karo, Mwaga/flush kwa choo ya shimo, Mwaga/flush mahali pengine, Mwaga/flush, lakini sijui ni mahali gani, Choo ya shimo iliyoboreshwa na iko na hewa ya kutosha, Choo ya shimo iliyo na saruji, Choo ya shimo isiyo na saruji/ shimo wazi, Choo ya mbolea, Choo ya ndoo, Hakuna kituo/kichaka/uwanja, Mengine |
| DHSHC107A | G11. Specify Other: | G11. Taja mengine: | ${DHSHC107} = 'Other' | | | |
| DHSHC108 | G12. Do you share this toilet facility with other households? | G12. Huwa mnatumia choo hii na nyumba zingine? | | yes | Yes, No, Refuse | Ndio, La, Kataa |
| DHSHC109 | G13. How many households use this toilet facility? | G13. Ni nyumba ngapi hutumia choo hii? | ${DHSHC108} = 'Yes' and not(${DHSHC107} = 'No_Facility/Bush/Field') | yes | Less Than 10 Households, 10 or More Households, Don't Know | Chini ya nyumba kumi, Nyumba kumi au zaidi, Sijui |
| DHSHC109A | G14. Please enter the number of households that use this toilet facility. | G14. Tafadhali weka idadi ya nyumba ambazo hutumia choo hii. | ${DHSHC109} = 'Less_than_10_households' | yes | | |
| DHSHC110 | G15. Does your household have any of the following? Select all that apply. | G15. Je, nyumba yako inayo yafuatayo? Chagua zote zifaayo. | | yes | Electricity, Radio, Television, Mobile Television, Non-mobile television, Refrigerator, Solar Panel, Table, Chair, Sofa, Bed, Cupboard, Clock, Microwave Oven, DVD Player, Cassette/CD Player | Stima, Redio, Televisheni, Televisheni ya mkono, Mashirika ya televisheni ya mkononi, Friji/jokofu/sanduku la barafu, Jopo nguvu ya jua/sola , Meza, Kiti, Kiti ya sofa, Kitanda, Kabati, Saa, Microwave, Mkanda (DVD player), Mkanda (CD player) |
| DHSHC111 | G16. What type of fuel does your household mainly use for cooking? | G16. Huwa unatumia njia gani ya kupika kwako nyumbani ? | | yes | Electricity, LPG/Natural Gas, Biogas, Parafin/Kerosene, Coal, Lignite, Charcoal, Wood, Straw/Shrubs/Grass, Agricultural Crop, Animal Dung, No Food Cooked in Household, Other | Stima, Gesi kiasili, Gesi ya kupika inayotengenezwa kutoka kwa samadi ya ng'ombe/biogas, Mafuta ya taa, Makaa ya mawe, Makaa, Kuni, Nyasi, Zao ya kilimo, Samadi ya mnyama, Hakuna chakula hupikwa nyumbani, Njia zingine |
| DHSHC111A | G17. 0 | G17. Eleza hizo njia zingine: | ${DHSHC111} = 'Other' | yes | | |
| DHSHC112 | G18. Is the cooking usually done in the main house, in a separate building, or outdoors? | G18. Upishi hufanyika kwa nyumba kuu, nyumba nyingine kando ama nje ya nyumba? | not(${DHSHC111} = 'No_Food_Cooked_in_Household') | yes | In the House, In a Separate Building, Outdoors, Other | Kwa nyumba, Nyumba ingine (jikoni ya nje), Nje ya nyumba, Kwingine |
| DHSHC112A | G19. 0 | G19. Eleza wapi kwingine: | ${DHSHC112} = 'Other' | | | |
| DHSHC113 | G20. Do you have a separate room which is used as a kitchen? | G20. Uko na chumba kingine kwenye nyumba yako yenye hutumika kama jikoni? | ${DHSHC112} = 'In_the_house' | yes | Yes, No, Refuse | Ndio, La, Kataa |
| DHSHC114 | G21. What is the main material of the floor in your household? | G21. Nini hutumika kutengeneza sakafu ya nyumba yako? | | yes | Earth/Sand, Dung, Wood Planks, Palm/Bamboo, Stone with Mud, Vinyl or Asphalt Strips, Ceramic Tiles, Cement, Carpet, Stone with Lime/Cement, Bricks, Other | Udongo/mchanga, Samadi ya ng'ombe, Mbao ya mti, Kiganja/mianzi, Mawe na matope, Lami iliyotandazwa, Tofali ya kauri (tiles), Saruji, Zulia, Mawe na saruji/mawe na chokaa, Tofali, Njia zingine |
| DHSHC114A | G22. 0 | G22. Eleza hizo njia zingine: | ${DHSHC114} = 'Other' | | | |
| DHSHC115 | G23. What is the main material of the roof in your household? | G23. Nini hutumika kutengeneza paa ya nyumba yako? | | yes | No Roof, Thatch/Grass/Makuti, Dung/Mud/Sod, Iron Sheets, Tin Cans, Stone with Mud, Concrete, Tiles, Stone with Lime/Cement, Bricks, Other | Haina paa, Nyasi, Matope, Mabati, Debe la kopo, Mawe na matope, Saruji, Tiles, Mawe na saruji/mawe na chokaa, Tofali, Njia zingine |
| DHSHC115A | G24. 0 | G24. Eleza hizo njia zingine: | ${DHSHC115} = 'Other' | | | |
| DHSHC116 | G25. What is the main material of the external walls in your house? | G25. Nini hutumika kutengeneza ukuta wa nyumba yako? | | yes | No Walls, Cane/Palm/Trunks, Dung/Mud/Sod, Bamboo with Mud, Stone with Mud, Uncovered Adobe, Plywood, Cardboard, Reused Wood, Iron Sheets, Cement, Stone with Lime/Cement, Bricks, Cement Blocks, Covered Adobe, Wood Planks/Shingles , Other | Haina ukuta, Miwa/kiganja/shina la mti, Matope/Samadi ya ng'ombe/Nyasi, Mianzi (bamboo) na matope, Mawe na matope, Matofali ya matope iliyowazi, Hadibodi, Kadibodi, Miti yenye imetumika tena, Mabati, Saruji, Mawe na saruji/mawe na chokaa, Mawe ya matofari, Mawe ya simiti, Matofali ya matope iliyofunikwa, Mbao ya mti, Njia zingine |
| DHSHC116A | G26. 0 | G26. Eleza hizo njia zingine: | ${DHSHC116} = 'Other' | | | |
| DHSHC117 | G27. How many rooms in this household are USED for sleeping? | G27. Ni vyumba ngapi HUTUMIKA kama vyumba vya kulala kwa hii nyumba yako? | | yes | #N/A | #N/A |
| DHSHC118 | G28. Does any member in this household own any of the following? Select all that apply. | G28. Kuna mtu yeyote mwenye anamiliki/ako na vitu vifuatavyo? Chagua zote zifaazo. | | yes | A watch, A bicycle, A Motorcycle or Motor Scooter, An Animal Drawn Cart, A Car or Truck, A Boat With a Motor, None of the above | Saa, Baiskeli, Pikipiki, Mkokoteni inayovutwa na punda, Gari, Mashua/boat ya mashine, Hakuna kati ya hayo juu |
| DHSHC119 | G29. Does any member of this household own any agricultural land? | G29. Kuna mtu yeyote wa nyumba hii mwenye anamiliki/ako na shamba la kilimo? | | yes | Yes, No, Refuse | Ndio, La, Kataa |
| DHSHC120 | G30. Do you know how many acres of land members of your household own? | G30. Je, unajua ni eka ngapi ya shamba familia yako inamiliki/iko nayo? | ${DHSHC119} = 'Yes' | yes | Yes, No, Refuse | Ndio, La, Kataa |
| DHSHC120A | G31. Please enter the number of acres. | G31. Tafadhali weka nambari ya ekari: | ${DHSHC120} = 'Yes' | yes | | |
| DHSHC121 | G32. Does this household own any livestock, herds, other farm animals, or poultry? | G32. Je, hii nyumba inamiliki/iko na mifugo yoyote, ng'ombe, wanyama wengine wa shamba ama kuku? | | yes | Yes, No, Refuse | Ndio, La, Kataa |
| DHSHC122 | G33. How many local cattle (indigenous) does this household own? | G33. Ni ngombe wangapi wa kienyeji wenye wanamilikiwa/wako kwa hili boma? | ${DHSHC121} = 'Yes' | yes | None, 1 to 4, 5 to 9, 10 or more | Hakuna, 1 hadi 4, 5 hadi 9, 10 au zaidi |
| DHSHC122A | G34. How many exotic/grade cattle does this household own? | G34. Ni ngombe wangapi wa kigeni wenye wanamilikiwa/wako kwa hili boma? | ${DHSHC121} = 'Yes' | yes | None, 1 to 4, 5 to 9, 10 or more | Hakuna, 1 hadi 4, 5 hadi 9, 10 au zaidi |
| DHSHC122B | G35. How many horses, donkeys, or camels does this household own? | G35. Ni farasi, punda au ngamia wangapi wenye wanamilikiwa/wako kwa hili boma? | ${DHSHC121} = 'Yes' | yes | None, 1 to 4, 5 to 9, 10 or more | Hakuna, 1 hadi 4, 5 hadi 9, 10 au zaidi |
| DHSHC122C | G36. How many goats does this household own? | G36. Ni mbuzi wangapi wanamilikiwa/wako kwa hili boma? | ${DHSHC121} = 'Yes' | yes | None, 1 to 4, 5 to 9, 10 or more | Hakuna, 1 hadi 4, 5 hadi 9, 10 au zaidi |
| DHSHC122D | G37. How many sheep does this household own? | G37. Ni kondoo wangapi wanamilikiwa/wako kwa hili boma? | ${DHSHC121} = 'Yes' | yes | None, 1 to 4, 5 to 9, 10 or more | Hakuna, 1 hadi 4, 5 hadi 9, 10 au zaidi |
| DHSHC122E | G38. How many chickens does this household own? | G38. Ni kuku wangapi wanamilikiwa/wako kwa hili boma? | ${DHSHC121} = 'Yes' | yes | None, 1 to 4, 5 to 9, 10 or more | Hakuna, 1 hadi 4, 5 hadi 9, 10 au zaidi |

| | | | | | | |
|---|---|---|---|---|---|---|
| DHSHC123 | G39. Does any member of this household have a bank account? | G39. Kuna mtu yeyote wa hii nyumba mwenye anayo akaunti ya benki? | | yes | Yes, No, Don't Know, Refuse | Ndio, La, Sijui, Kataa |
| DHSHC124 | G40. At any time in the past 12 months, has anyone come into your dwelling to spray the interior walls against mosquitos? | G40. Kwa wakati wowote chini ya miezi 12, kuna mtu yeyote amekuja kwa makao yako kufukiza kuta za ndani dhidi ya mbu? | | yes | Yes, No, Don't Know, Refuse | Ndio, La, Sijui, Kataa |
| DHSHC126 | G41. Does your household have any mosquito nets that can be used while sleeping? | G41. Je, nyumba yako inayo neti ya kuzuia mbu yenye yaweza kutumika wakati wa kulala? | | yes | Yes, No, Refuse | Ndio, La, Kataa |
| DHSHC127 | G42. How many mosquito nets does your household have? If 7 or more nets, record '7'. | G42. Nyumba yako iko na neti ngapi ya kuzuia mbu? Kama ni saba au zaidi, andika saba. | ${DHSHC126} = 'Yes' | yes | | |
| DHSRB104 | G43. Have you ever attended school? | G43. Umewahi kwenda shule? | | yes | Yes, No, Refuse | Ndio, La, Kataa |
| DHSRB105 | G44. What is the highest level of school you attended? | G44. Ulifika kiwango gani cha juu cha masomo? | ${DHSRB104} = 'Yes' | yes | Primary, Secondary/'A' level, Post-primary/Vocational, College (Middle Level), University | Msingi/Primary, Sekondari/Shule ya upili, Shule ya ufundi/Technical , Chuo/College , Chuo kikuu/University |
| DHSRB106 | G45. What is the highest standard/form/year you completed at that level? IF YOU COMPLETED LESS THAN ONE YEAR AT THAT LEVEL, RECORD '0'. | G45. Ulifika (darasa/kidato/mwaka) gani katika kiwango hicho? KAMA ULIMALIZA CHINI YA MWAKA MOJA KWA KIWANGO HICHO, REKODI '0' | ${DHSRB104} = 'Yes' | yes | | |
| DHSRB110 | G46. Do you read a newspaper or magazine at least once a week, less than once a week or not at all? | G46. Huwa unasoma gazeti hata mara moja kwa wiki, chini ya wiki moja ama husomi kamwe? | | yes | At Least Once a Week, Ocasionally/Once in a While, Not at All | Angalau mara moja kwa wiki , Mara moja moja, Kamwe |
| DHSRB111 | G47. Do you listen to the radio at least once a week, less than once a week or not at all? | G47. Huwa unaskiliza redio hata mara moja kwa wiki, chini ya mara moja kwa wiki ama huskizi kamwe? | | yes | At Least Once a Week, Ocasionally/Once in a While, Not at All | Angalau mara moja kwa wiki , Mara moja moja, Kamwe |
| DHSRB112 | G48. Do you watch television at least once a week, less than once a week or not at all? | G48. Huwa unatazama televisheni hata mara moja kwa wiki, chini ya mara moja kwa wiki ama hutazami kamwe? | | yes | At Least Once a Week, Ocasionally/Once in a While, Not at All | Angalau mara moja kwa wiki , Mara moja moja, Kamwe |
| DHSRB113 | G49. What is your religion? | G49. Wewe ni wa dini gani? | | yes | Roman Catholic, Protestant or other Christian , Muslim, No religion, Other | Catoliki, Kiprotestanti (Christ-Co, Winner's Chapel, Friends/Quakers, PAG, AIC, Divine, Deliverance n.k.) , Muislamu, Hauna dini, Kabila nyingine |
| DHSRB113A | G50. Specify Other: | G50. Taja hiyo dini nyingine: | ${DHSRB113} = 'Other' | | | |
| DHSRB114 | G51. What is your ethnic group/tribe? | G51. Wewe ni wa kabila gani? | | yes | Embu, Kalenjin, Kamba, Kikuyu, Kisii, Luhya , Luo, Maasai, Meru, Mijikenda/Swahili, Somali, Taita Taveta, Other | Embu, Kalenjin, Kamba, Kikuyu , Kisii, Luhya, Luo, Maasai, Meru, Mijikenda/Swahili, Somali, Taita Taveta, Njia zingine |
| DHSRB114A | G52. Specify Other: | G52. Taja nyingine: | ${DHSRB114} = 'Other' | | | |
| DHSRB108 | G53. Thank you for completing this portion of the survey. Please return the tablet to the enumerator for the final question. (111) | G53. Asante kwa kumaliza sehemu hii ya utafiti. Tafadhali rudisha kibao simu kwa mtafiti akuulize swali la mwisho. (111). | | yes | #N/A | #N/A |
| DHSRB108A | G54. The child is reading a book | G54. Huyu mtoto anasoma kitabu. | | | | |
| DHSRB108B | G55. Reading Capacity: | G55. Uwezo wa kusoma: | | yes | Cannot Read at All, Able to Read Only Parts of Sentence, Able to Read Whole Sentence, No Card with Required Language, Blind/Visually Impaired | Hawezi soma kamwe, Huweza kusoma tu sehemu ya sentensi, Huweza kusoma sentensi yote, Hakuna lugha inayoeleweka, Kipofu/Haoni vizuri |
| DHSRB108C | G56. Specify Language: | G56. Taja lugha: | ${DHSRB108B} = 'No_Card_With_Required_Language' | yes | English, Swahili | English, Swahili |
| New47 | G57. Did the participant answer the survey questions by listening to the enumerator administer the questions or answering on her own? | G57. Did the participant answer the survey questions by listening to the enumerator administer the questions or answering on her own? | | yes | | |
| New49 | G58. In which language was the screening completed? | G58. In which language was the screening completed? | | yes | | |
| New18 | G59. End of Survey | G59. Mwisho wa Utafiti | | | | |
| endHH | | | | | | |
| beginConsent | | | ${unmetNeed}=1 | | | |
| New40 | H7. Did this participant sign the consent form? | H7. Did this participant sign the consent form? | | yes | | |
| New46 | H8. [Text XXXXX: keyword +254XXXXXXXXX.] | H8. [Text XXXXX: keyword +254XXXXXXXXX.] | ${New40}='No' | | | |
| IDpart | H9. [Text XXXXX: keyword +254XXXXXXXXX.] Fill out the financial record book.] Please enter the participant ID sent to you by SMS: | H9. [Text XXXXX: keyword +254XXXXXXXXX.] Fill out the financial record book.] Please enter the participant ID sent to you by SMS: | ${New40}='Yes' | yes | | |
| endConsent | | | | | | |
| Idrecord | I1. Please fill out the financial record book and enter the form ID from the book (T_-XXXX): | I1. Please fill out the financial record book and enter the form ID from the book (T_-XXXX): | ${passedEligibility1}=0 or ${passedEligibility2}=0 or ${passedEligibility3}=0 or ${unmetNeed}=0 or ${New40}='No' | yes | | |

follow-up survey



| name | label::English | label::Swahili | relevant | required | options.english | options.swahili |
|---|---|---|---|---|---|---|
| start | A1. Start Time | A1. Start Time | | yes | | |
| end | A2. End Time | A2. End Time | | yes | | |
| today | A3. Date of Survey | A3. Date of Survey | | yes | | |
| deviceid | A4. Device | A4. Device | | yes | | |
| beginIdentification | | | | | | |
| IDPart | B1. [Enter the Participant ID.] | B1. [Enter the Participant ID.] | | yes | | |
| participant | B2. [Select participant] | B2. [Select participant] | | yes | | |
| partName | if(string-length(${participant}) != 0,jr:choice-name(${participant},'${participant}'),'(not a valid participant)') | | | | | |
| Displaypart | B4. [You chose ${partName}. If that is not correct, swipe back.] | B4. [You chose ${partName}. If that is not correct, swipe back.] | | | | |
| Pullassign | if(${IDPart}=XXX or ${IDPart}=XXX or ..., 'control', 'treatment') | | | | | |
| ID2 | B9. [What is the reason for calling the participant?] | B9. [What is the reason for calling the participant?] | | yes | Participant prefers a follow up call, Something went wrong with her survey, No reply to SMS invite | Participant prefers a follow up call, Something went wrong with her survey, No reply to SMS invite |
| ID3 | B10. [Did the call connect?] | B10. [Did the call connect?] | | yes | Yes, No | Ndio, La |
| ID4 | B11. Hi, I'm [MY NAME] from the Bungoma County Women's Health Study. Is this ${partName}? | B11. Jambo, jina langu ni [Jina] kutoka utafuti wa Afya ya Wanawake kutoka Bungoma. Wewe ndiye ${partName}? | ${ID3}='Yes' | | Yes, No | Ndio, La |
| ID5 | B12. [Is this the correct participant?] | B12. [Is this the correct participant?] | ${ID3}='Yes' | yes | Yes, No | Ndio, La |
| FUP43 | B13. Is ${partName} available to speak right now? | B13. Je ${partName} yuko tayari kuzungumza wakati huu? | ${ID5}='No' | yes | Yes, No | Ndio, La |
| FUP44 | B14. Great, Can you please put her on the phone? | B14. Sawa, unaweza kumweka kwenye simu ili azungumze? | ${FUP43}='Yes' | yes | Yes, No | Ndio, La |
| FUP45 | B15. Ok, I'll try again another time this week. | B15. Sawa, nitajaribu tena kuzungumza nawe wakati mwengine wiki hii. | ${FUP43}='No' or ${FUP44}='No' | | | |
| FUP46 | B16. Hi, I'm [MY NAME] from the Bungoma County Women's Health Study. | B16. Jambo, mimi ni [Jina] kutoka utafiti ya afya ya wanawake kutoka Bungoma. | ${FUP44}='Yes' | | | |
| Assign | | | ${ID5}='Yes' or ${FUP44}='Yes' | | | |
| endIdentification | | | | | | |
| beginIntro | | | ${ID5}='Yes' or ${FUP44}='Yes' | | | |
| FUP1 | C1. I received a message that you would prefer a follow up call. I'm here to help. This follow-up survey should only take 5 minutes. When you're done, I'll send you Ksh 200 airtime as a thank you. Do you have time? | C1. Nimepata ujumbe kwamba ungependa kufuatiliwa kwa njia ya kupigiwa simu. Niko hapa kukusaidia.Uchunguzi huu utachukua dakika tano tu. Utakapomaliza tutakutumia shilingi mia mbili ya pesa za kuzungumza kwa simu. Je una wakati? | ${ID2}='Prefer' | yes | Yes, No | Ndio, La |
| FUP48 | C2. Thanks again for joining our study! I'd like to invite you to participate in a very short follow-up. It should take you less than 5 minutes. When you're done, I'll send you Ksh 200 airtime as a thank you. Do you have time? | C2. Ahsante kwa kuungana nasi katika utafiti huu. Ningependa kukualika uhudhurie mfuatilio mfupi sana. Itachukua muda wa chini ya dakika tano. Utakapomaliza tutakutumia shilingi mia mbili ya pesa za kuzungumza kwa simu. Je una wakati? | ${ID2}='No_Reply' | yes | Yes, No | Ndio, La |
| FUP37 | C3. I received a message that something went wrong in your survey. I'm here to help. This follow-up survey should only take 5 minutes. When you're done, I'll send you Ksh 200 airtime as a thank you. Do you have time? | C3. Nimepata ujumbe ya kwamba kuna jambo liloharibika wakati uchunguzi ulikua unafanyika. Niko hapa kukusaidia. Uchunguzi huu utachukua dakika tano tu. Utakapomaliza tutakutumia shilingi mia mbili ya pesa za kuzungumza kwa simu. Je una wakati? | ${ID2}='Wrong' | yes | Yes, No | Ndio, La |
| endIntro | | | | | | |
| beginMessages | begin group | | ${FUP1}='Yes' or ${FUP37}='Yes' | | | |
| FUP38 | D1. Let's get started! I'll ask some questions. There are no right or wrong answers. | D1. Tuanze! Nitauliza maswali chache. Hakuna swali sahihi au kosa. | | | | |
| FUP2 | D2. When you completed our first survey in the market, you said you were not using any form of family planning. How about now? Are you currently doing something or using any method to delay or avoid getting pregnant? | D2. Ulipokamilisha utafiti wetu wa maswali ya upangaji uzazi ulisema ya kwamba hautumii njia yoyote ya upangaji uzazi. Kwa sasa, unatumia mbinu zozote kuchelewa ama kuepuka kupata/kushika mimba? | | yes | Yes, No | Ndio, La |
| FUP3 | D3. So you are not currently using any kind of family planning, like pills, injections, or implants. Is that correct? | D3. Kwa hivyo hautumii njia yoyote ya upangaji uzazi, kama tembe, sindano ama kidude. Hio ni sawa? | ${FUP2}='No' | yes | Correct, Incorrect | Sahihi, Si sahihi |
| FUP4 | D4. Have you used any forms of family planning since the first election in August, even if you have since stopped? | D4. Umetumia njia yoyote ya upangaji uzazi tangu uchaguzi wa kwanza katika mwezi wa nane, hata iwapo uliwacha kitambo? | ${FUP3}='Correct' | yes | Yes, No | Ndio, La |
| FUP5 | D5. Which method? | D5. Ni mbinu gani unayotumia? | ${FUP2} = 'Yes' or ${FUP3}='Incorrect' or ${FUP4}='Yes' | yes | | |
| FUP6 | D6. Are you satisfied with this method so far? | D6. Umeridhika na njia hii ya upangaji uzazi? | ${FUP2}='Yes' or ${FUP3}='Incorrect' | yes | Yes, No | Ndio, La |
| FUP7 | D7. Sorry to hear that. Why not? | D7. Pole sana, kwa nini haujaridhika? | ${FUP6}='No' | yes | | |
| FUP8 | D8. Have you visited a family planning provider for any reason since the first election in August? | D8. Umemtembelea mhudumu wa upangaji uzazi kwa sababu yoyote tangu uchaguzi wa kwanza katika mwezi wa nane? | | yes | Yes, No | Ndio, La |
| FUP9 | D9. Which provider? | D9. Ni kituo gani cha afya uliyotembelea? | ${FUP8}='Yes' | yes | | |
| FUP10 | D10. When did you go? | D10. Ulienda lini? | ${FUP8}='Yes' | yes | | |
| FUP11 | D11. Did you have to pay any money at this visit? | D11. Je, ulilipia huduma hiyo? | ${FUP8}='Yes' | yes | Yes, No | Ndio, La |
| FUP12 | D12. How much? | D12. Pesa ngapi? | ${FUP11}='Yes' | yes | | |
| FUP13 | D13. A while back we invited you to try a new phone service called Nivi. Have you ever called Nivi to try the service? | D13. Kitambo kidogo tuliweza kukualika ili utumia huduma mpya ya simu inayoitwa Nivi . Je umewahi kupigia Nivi simu ili kujaribu huduma hii? | ${Assign}=1 | yes | Yes, No | Ndio, La |
| FUP30 | D14. Nivi is a new phone service that helps women access family planning? Have you ever heard of it? | D14. Nivi ni huduma mpya ya simu ambayo inasaidia wanawake kuweza kupata huduma ya upangaji uzazi. Je umewahi kusikia Nivi? | ${Assign}=0 | yes | Yes, No | Ndio, La |
| FUP31 | D15. OK, we'll send you an invite. You might like to give it a try! | D15. Sawa, tutakutumia mwaliko. Unaweza kuijaribu! | ${FUP30}='No' | | | |
| FUP32 | D16. Have you ever called Nivi to try the service? | D16. Je, umewahi kupigia simu Nivi ili kujaribu huduma hii? | ${FUP30}='Yes' | yes | Yes, No | Ndio, La |

| | | | | | | |
|---|---|---|---|---|---|---|
| FUP14 | D17. Please enter the phone number you used when trying Nivi. | D17. Tafadhali andika nambari yako uliotumia ukijaribu Nivi. | ${FUP32}='Yes' or ${FUP13}='Yes' | yes | | |
| FUP15 | D18. Did you complete the Nivi screening and receive a referral code via SMS? | D18. Je uliweza kukamilisha uchunguzi wa Nivi na kupata kodi ya rufaa kupitia ujumbe mfupi? | ${FUP32}='Yes' or ${FUP13}='Yes' | yes | Yes, No | Ndio, La |
| FUP16 | D19. Did you like using Nivi? | D19. Je ulipenda kutumia Nivi? | ${FUP32}='Yes' or ${FUP13}='Yes' | yes | Yes, No | Ndio, La |
| FUP17 | D20. That's it! Thanks for taking the time to answer a few questions. I'll send some appreciation. We'll be back in touch in a few months. Bye! | D20. Ni hayo tu kwa sasa. Asante kwa kuchukua muda wako kujibu maswali haya. Nitatuma shukrani zangu. Tutaweza kuzungumza baada ya miezi michache. Kwaheri! | | | | |
| FUP42 | D21. [Did you speak to the participant in Swahili or English?] | D21. [Did you speak to the participant in Swahili or English?] | | yes | Swahili, English | Swahili, English |
| endMessages | | | | | | |
| beginOther | begin group | | ${FUP1}='No' or ${FUP37}='No' or ${FUP48}='No' | | | |
| FUP39 | E1. When would be a good day for me to call you back? | E1. Ni siku gani itakayokuwa nzuri ya kukupigia? | | yes | | |
| FUP40 | E2. Should I call you in the morning, afternoon, or evening? | E2. Je, ninaweza kukupigia asubuhi, mchana, au jioni? | | yes | Morning, Afternoon, Evening | Asubuhi, Mchana, Jioni |
| FUP41 | E3. Thanks, I'll call back then. Have a nice day! | E3. Asante, nitakupigia simu. Uwe na siku njema! | | | | |
| endOther | | | | | | |
| FUP47 | F1. [Was the follow-up survey completed?] | F1. [Was the follow-up survey completed?] | | yes | Yes, No | Ndio, La |
