# Supplementary material for: Developing a Digital Marketplace for Family Planning: Pilot Randomized Encouragement Trial
Source: J Med Internet Res. 2018 Jul 31;20(7):e10756. doi: 10.2196/10756 (PMC6092593; doi:10.2196/10756)
Supplement: Multimedia Appendix 2 [file jmir_v20i7e10756_app2.pdf]

Appendix C

Unmet Need

Algorithm based on DHS guidelines and informed by Bradley et al. (2014).

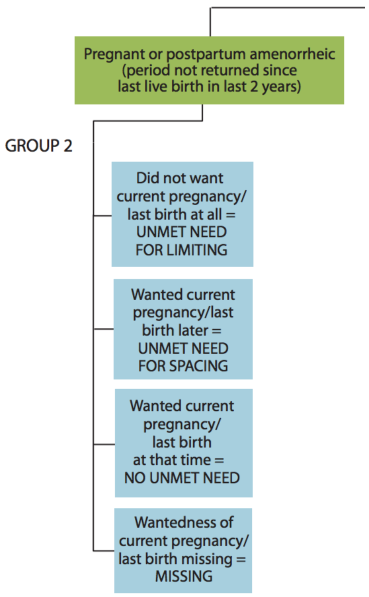

group2:

```
if(
${New19}='Less_Than_2_Years_Ago' and   # birth less than 2 years ago (not necessarily live)
(${DHSR238}=='Between_24_and_60_months' or   # period more than 2 years
 ${DHSR238}='More_than_60_months'),
1, # means in group 2
0)
```

group2Need:

```
if(
${DHSR228}='Yes', # wanted last pregnancy
'no_unmet_need',  # means no unmet need
'unmet_need')
```

group2UnmetType:

```
if(
${DHSR229}='Later', # wanted later
'spacing',          # means unmet need for spacing
'limiting')
```

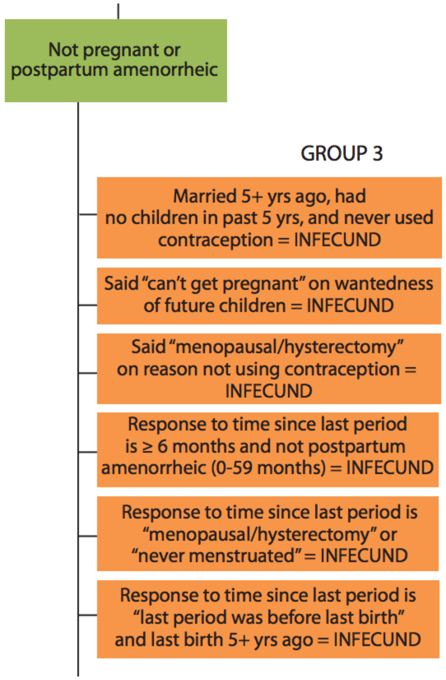

group3:

```
if(
((${New21}='Yes' and ${DHSC313}="No" and # married 5+ years ago, never used contraception
 (${DHSR201}='No' or ${New19}='More_Than_5_Years_Ago')) or # no kids or none past 5 years
${DHSFP704}='I_Cant_Get_Pregnant' or     # can't get pregnant
${DHSR238}='menopause_hysterectomy' or    # menopause or hysterectomy
${DHSR238}='More_than_60_months') and     # no period for 5 years (simplified here)
${group2}!=1, # not in group 1 (should be redundant)
1,            # means in group 3
0)
```

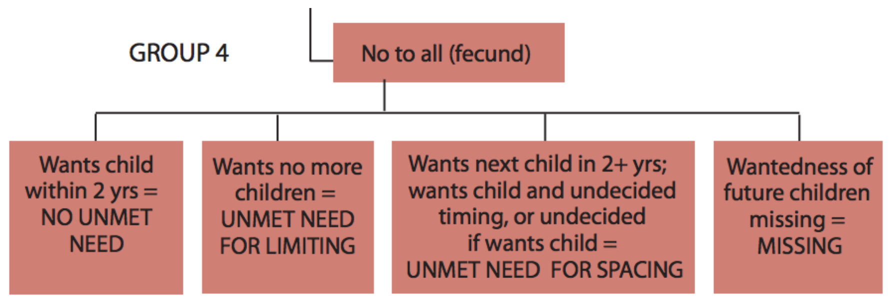

group4:

```
if(
${group3!=1,   # not group 3 is also not group 2 based on group 3 rules
1,             # means in group 4
0)
```

group4Need:

```
if(
${DHSFP705}='Soon/Now' or ${DHSFP705}='within_2_years',  # wants kids < 2 years
'no_unmet_need', # means no unmet need
'unmet_need')
```

group4UnmetType:

```
if(
${DHSFP704}='No_More/None', # wants no more kids
'limiting', # means limiting
'spacing')
```

<u>Unmet Need:</u>

Extension to women not married/in-union

```
if(
${DHSMSA615}='within_last_month', # sexually active in last month
1, # means yes
0)
```

Logic:

```
if(
(${group2Need}='unmet_need' or ${group4Need}='unmet_need') and, # unmet need in groups 2/4
${singleActive}!=0,
1, # means unmet need
0)
```
