# Supplementary material for: Developing a Digital Marketplace for Family Planning: Pilot Randomized Encouragement Trial
Source: J Med Internet Res. 2018 Jul 31;20(7):e10756. doi: 10.2196/10756 (PMC6092593; doi:10.2196/10756)
Supplement: Multimedia Appendix 3 [file jmir_v20i7e10756_app3.pdf]

Appendix A

Table A1

Impact on contraception adoption, sample limited to found at follow-up

	First stage Tried intervention	*ITT estimation* Adopted contraception	*IV estimation* Adopted contraception
	(1)	(2)	(3)
Assigned to treatment	0.31^{***}	0.20	
	(0.19, 0.44)	(−0.07, 0.47)	
Tried intervention			0.44
			(−0.15, 1.03)
Mean in control group	0.02	0.43	
Includes controls	Yes	Yes	Yes
Observations	112	50	50

Notes: The first stage regression estimate (Column 1) is the coefficient on assignment to treatment from an OLS regression of intervention use on assignment. The intent-to-treat (ITT) estimate (Column 2) is the coefficient on assignment to treatment from an OLS regression of contraception adoption on assignment. The instrumental variables (IV) estimate (Column 3) is the coefficient on intervention use in a two-stage least squares regression of contraception adoption on assignment and intervention use. Controls include an indicator for mode of follow-up survey administration and several baseline characteristics, including: age, number of children born, and indicators for having attended post-secondary schooling, past use of family planning, being married or in a union, and nulligravida. Corrected Huber-White standard errors.

$^{*}p<0.1$; $^{**}p<0.05$; $^{***}p<0.01$

Table A2

Impact on contraception adoption, probit regression

	First stage Tried intervention	*ITT estimation* Adopted contraception	*IV estimation* Adopted contraception
	(1)	(2)	(3)
Assigned to treatment	1.71***	0.68**	
	(0.54, 2.89)	(0.03, 1.34)	
Tried intervention			2.21*
			(-0.08, 4.5)
Mean in control group	0.02	0.43	
Includes controls	Yes	Yes	Yes
Observations	112	112	112

Notes: The first stage regression estimate (Column 1) is the coefficient on assignment to treatment from a probit regression of intervention use on assignment. The intent-to-treat (ITT) estimate (Column 2) is the coefficient on assignment to treatment from a probit regression of contraception adoption on assignment. The instrumental variables (IV) estimate (Column 3) is the coefficient on intervention use in a probit regression of contraception adoption on assignment and intervention use (run in Stata MP 12, Newey's two-step estimator). Controls include an indicator for mode of follow-up survey administration and several baseline characteristics, including: age, number of children born, and indicators for having attended post-secondary schooling, past use of family planning, being married or in a union, and nulligravida.

*p<0.1; **p<0.05; ***p<0.01
